# Altering the fatty acid profile of *Yarrowia lipolytica* to mimic cocoa butter by genetic engineering of desaturases

**DOI:** 10.1186/s12934-022-01748-x

**Published:** 2022-02-19

**Authors:** Oliver Konzock, Yuika Matsushita, Simone Zaghen, Aboubakar Sako, Joakim Norbeck

**Affiliations:** Division of Systems and Synthetic Biology, Department of Biology and Biological Engineering, Chalmers University of Technology, Göteborg, Sweden

**Keywords:** Food oil, Microbial oil, Triglycerides, Phospholipids, Growth performance, Lipid profile

## Abstract

**Background:**

Demand for Cocoa butter is steadily increasing, but the supply of cocoa beans is naturally limited and under threat from global warming. One route to meeting the future demand for cocoa butter equivalent (CBE) could be to utilize microbial cell factories such as the oleaginous yeast *Yarrowia lipolytica.*

**Results:**

The main goal was to achieve triacyl-glycerol (TAG) storage lipids in *Y. lipolytica* mimicking cocoa butter. This was accomplished by replacing the native Δ9 fatty acid desaturase (Ole1p) with homologs from other species and changing the expression of both Ole1p and the Δ12 fatty acid desaturase (Fad2p). We thereby abolished the palmitoleic acid and reduced the linoleic acid content in TAG, while the oleic acid content was reduced to approximately 40 percent of the total fatty acids. The proportion of fatty acids in TAG changed dramatically over time during growth, and the fatty acid composition of TAG, free fatty acids and phospholipids was found to be very different.

**Conclusions:**

We show that the fatty acid profile in the TAG of *Y. lipolytica* can be altered to mimic cocoa butter. We also demonstrate that a wide range of fatty acid profiles can be achieved while maintaining good growth and high lipid accumulation, which, together with the ability of *Y. lipolytica* to utilize a wide variety of carbon sources, opens up the path toward sustainable production of CBE and other food oils.

**Supplementary Information:**

The online version contains supplementary material available at 10.1186/s12934-022-01748-x.

## Introduction

Cocoa butter is one of the main ingredients in chocolate and is additionally used in other products such as cosmetics. Cocoa butter mainly consists of triacylglycerol (TAG, three fatty acids attached to a glycerol backbone) and is produced from cocoa tree seeds (*Theobroma cacao)*. Originally native to Central America, the growing area has spread to almost all tropical countries of the world [1]. The cocoa tree needs 5 years to bear fruits and about ten years to reach its maximum yield. Additionally, it only grows in the tropical *cocoa belt* between 10° and 20° north to south [1]. There is a natural fluctuation in production, which, combined with rising pressure from pests, disease [2], and global warming, is projected to impact the cocoa production areas [3], which might cause shortages in the future.

A solution to counterbalance fluctuation and sustainably prevent supply shortfalls is producing cocoa butter equivalents (CBE) in microbial cell factories, such as yeast. CBE are lipids with similar properties as cocoa butter and can be blended with natural cocoa butter [4]. The fatty acid profile of cocoa butter mainly consists of palmitic acid (C16:0, 26.2%), stearic acid (C18:0, 35.8%) and oleic acid (C18:1, 33.6%), and a small amount of linoleic acid (C18:2, 2.7%) [5]. Multiple studies have demonstrated cocoa butter equivalent production in the model organism *Saccharomyces cerevisiae* [4, 6, 7]. However, *S. cerevisiae* does not have a high native lipid accumulation capacity, limiting its productivity and the economic feasibility of the process [4].

A more promising type of cell factory to produce lipids and lipid derivates are oleaginous yeasts, which are characterized by having at least 20% of their cell dry weight as lipids, mainly constituted of TAGs [8]. Multiple oleaginous yeasts (e.g. Y*arrowia lipolytica*, *Rhodotorula toruloides*, *Trichosporon oleaginosus*) have potential as platform strains for CBE production and have recently been reviewed [9].

**Fig. 1:**
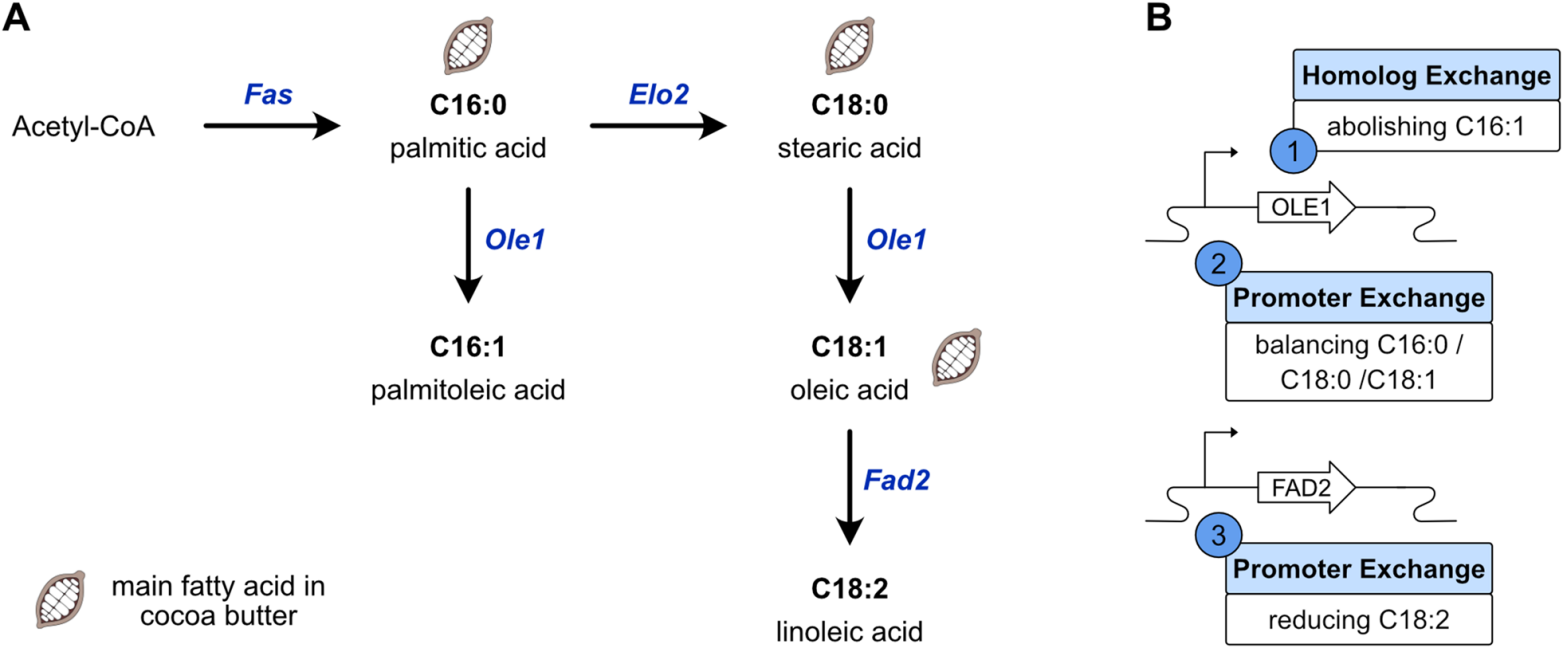
Overview of fatty acid synthesis in *Yarrowia *lipolytica and engineering strategy of the study. A The three main fatty acid species found in cocoa butter are marked with a cocoa pod. Names in blue display the proteins catalyzing the reaction. *Fas* fatty acid synthetase, *Elo2* fatty acid elongase, *Ole1* Δ9 fatty acid desaturase, *Fad2* Δ12 fatty acid desaturase. B The three main experimental strategies of this study: first, the native *OLE1* was exchanged with homologs to abolish palmitoleic acid (C16:1). Second, the native OLE1 promoter was exchanged to balance palmitic, stearic and oleic acid (C16:0, C18:0 and C18:1). Finally, the FAD2 promoter was exchanged to reduce the linoleic acid content (C18:2)

*Y. lipolytica* is the most studied oleaginous yeast and is generally regarded as safe (GRAS) [10]. Additionally, *Y. lipolytica* has a high tolerance to salt and acidic pH [11] and can be engineered to consume various carbon sources (e.g. pentoses such as xylose or lipids) [12]. The primary fatty acids found in *Y. lipolytica* are palmitic acid (C16:0), palmitoleic acid (C16:1), stearic acid (C18:0), oleic acid (C18:1), and linoleic acid (C18:2) [13]. De novo fatty acid synthesis produces palmitic acid (C16:0) and stearic acid (C18:0) that are bound to Coenzyme A and the Δ9 desaturase Ole1p can desaturate them to form palmitoleic acid (C16:1) and oleic acid (C18:1) [14]. The latter can be converted to phosphatidylcholine and further desaturated by the Δ12 desaturase Fad2p (Fig. 1A) [15]. Both desaturases are membrane-bound and receive electrons from NADH via an NADH-dependent cytochrome b_5_ reductase [16–18].

The primary aim of this study was to mimic the fatty acid profile of cocoa butter in the oleaginous yeast *Y. lipolytica* by genetic engineering of its desaturases. A secondary goal was to develop a palette of high lipid content strains with different fatty acid profiles to produce a selection of microbial oils with different properties. A previous study compared different oleaginous yeasts for their potential as a platform to produce cocoa butter-like lipids and found that the main drawback of *Y. lipolytica* was its high palmitoleic acid (C16:1) and linoleic acid (C18:2) content [19]. To overcome these drawbacks, we first exchanged its Ole1p with homologs from other organisms with low affinity for C16:0 fatty acids to reduce the cellular palmitoleic acid (C16:1) content (Fig. 1B). Then, we exchanged the native *OLE1* promoter to vary the expression level of the *OLE1* homologs and analyzed the fatty acid profiles to identify a strain with a similar FA profile as cocoa butter while maintaining a high lipid production. To further study the impact of different fatty acid profiles on the growth and lipid content, we also tested different expression levels of *FAD2*.

## Results

### Exchange of *OLE1* with homologs from *Arxula adeninivorans* (*AaOLE1*), *Gloeophyllum trabeum* (*GtOLE1*), and *Rhodotorula toruloides* (*RtOLE1*)

**Fig. 2:**
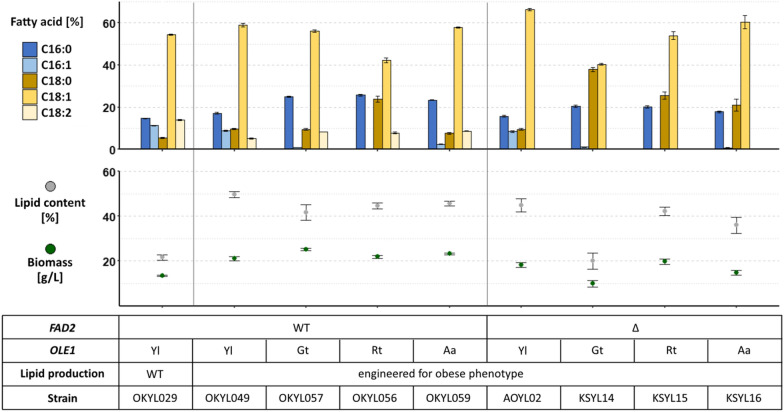
Replacement of native OLE1 with homologs in *FAD2* wild type and deletion strain. Strains were expressing homologs of Ole1p under the control of the native OLE1 promoter. Strains were cultivated for 96 h in LPU media, and the fatty acid profile was determined by FAME extraction. Lipid content represents g FAME per g cell dry weight. Displayed is the mean and standard error of n ≥ 4 replicates. Data for OKYL029 from [20]. *Yl* *Y. lipolytica*, *Gt* *G. trabeum*, *Rt* *R. toruloides*, *Aa* *A. adeninivorans*, *WT* wild type, *Δ* deletion

To establish a food oil production strain, it is crucial to alter its fatty acid profile while maintaining high lipid titers. We first compared the fatty acid composition of a non-engineered strain (OKYL029, mhy1Δ to avoid undesired pseudohyphal growth) [20] with a derived strain engineered for lipid production (OKYL049—deletion of sterol ester production (are1Δ) and increased TAG production (DGA1 overexpression)) [21], leading to a 2.5-fold increase in lipid content. We noted that OKYL049 displayed a decrease in linoleic acid (C18:2) and an increase in stearic acid (C18:0), both of which properties are desirable for CBE-production (Fig. 2). Therefore, all subsequent work was performed in the OKYL049 background.

Cocoa butter is largely devoid of palmitoleic acid (C16:1), and our first objective was to reduce the C16:1 content while maintaining good growth and lipid production. We chose to modulate the activity of the ∆9 desaturase Ole1p. As previously reported, the deletion of *OLE1* was not viable unless supplemented with unsaturated fatty acids [22] (Additional file 1: Fig. S1). During these experiments, we noted that even supplementation with heptadecenoic acid (C17:1), a fatty acid not naturally produced by *Y. lipolytica*, allowed growth (Additional file 1: Fig. S1). Instead of deleting *OLE1*, we exchanged the open reading frame of the native *YlOLE1* with codon-optimized homologs of *OLE1* from *Arxula adeninivorans* (*AaOLE1*), *Gloeophyllum trabeum* (*GtOLE*), and *Rhodotorula toruloides* (*RtOLE1*), all of which have been reported to have low affinity for C16 fatty acids [22]. The growth of these strains was largely unaffected (Fig. 2). As expected, we found that the palmitoleic acid content (C16:1) was highly reduced in all strains expressing any of the three *OLE1*-homologs (Fig. 2), supporting that the activity of all three homologs with palmitic acid (C16:0) as substrate is lower compared to the native *YlOLE1*. However, the strain expressing *AaOLE1* (OKYL059) showed clear, albeit low, levels of C16:1. The strain expressing *RtOLE1* (OKYL056) showed the most cocoa butter-like fatty acid profile (26% C16:0, 0% C16:1, 24% C18:0, 42% C18:1, 8% C18:2) (Fig. 2).

Cocoa butter is very low in linoleic acid (C18:2) content. In order to abolish this fatty acid, we deleted the gene encoding the Δ12 desaturase (*FAD2*) [23] in the above detailed strains expressing different *OLE1*-homologs. As expected, the FAD2-deletion abolished C18:2 from the fatty acid profiles (Fig. 2). The deletion of *FAD2* in the wild-type *OLE1* strain did not reduce growth or lipid production (AOYL02; Fig. 2). Among the *FAD2* deletion strains, KSYL14 (expressing *GtOLE1)* showed the fatty acid profile most similar to cocoa butter (20.5% C16:0, 1.1% C16:1, 38% C18:0, 40.4% C18:1). However, the biomass and lipid content of this strain were strongly reduced compared to the parental strain (AOYL02) (18 to 10 g/L biomass and 45 to 20% lipid content, respectively). Supplementing unsaturated fatty acids to the media recovered the growth defect of KSYL14 (Additional file 1: Fig. S3). In the strains expressing *RtOLE1* or *AaOLE1*, oleic acid (C18:1) proportion in the fatty acid profile was increased.

### Promoter exchange of *OLE1*

**Fig. 3:**
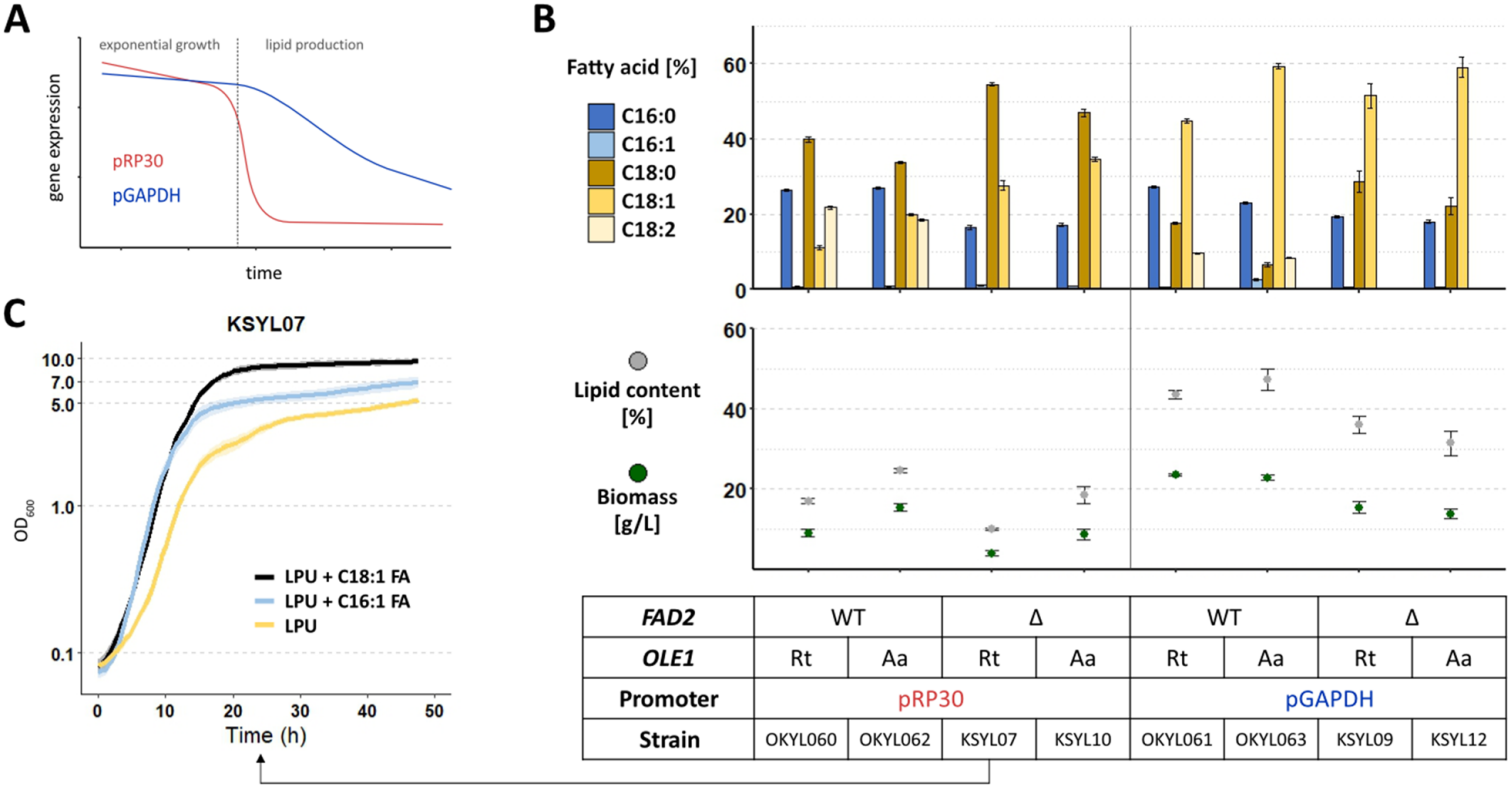
Promoter exchange of *OLE1* homologs. The native *OLE1* gene was exchanged for either *RtOLE1* or *AaOLE1*, and the native promoter was exchanged for either pGAPDH or pRP30. **A** Strains were cultivated for 96 h in LPU media, and the fatty acid profile was determined by FAME extraction. Lipid content represents g FAME per g cell dry weight. Displayed is the mean and standard error of n ≥ 4 replicates. **B** Scheme of expected promoter activity of pRP30 and pGAPDH based on data from [24]. Dashed line marks the onset of lipid production due to nitrogen limitation. **C** Growth curves of strain KSYL07. Cells were cultivated in 96-well plates in LPU media with or without the addition of palmitoleic acid (C16:1) or oleic acid (C18:1) (500 mg/L in 1% Tween 20), and OD_600_ was measured every 30 min. The lines and shadows represent the average and standard deviation of quadruplicates, respectively

All our engineered strains displayed a level of C18:1 that was too high compared to cocoa butter. Therefore, as a second step towards a tailored fatty acid profile, we decided to alter the expression level of the two *OLE1* homologs that showed the best results (i.e. highly reduced C16:1 content and no reduction in growth or lipid production) in the previous experiment (Fig. 2), *RtOLE1* and *AaOLE1*. This set of experiments aimed to have enough expression of the *OLE1* homologs to supply the cells with unsaturated fatty acids during exponential phase to maintain good growth, while having a minimal expression during the lipid production phase to reduce the amount of oleic acid (C18:1) in the final product. From published transcriptomics analyses [24], we identified several promoters that were hypothesized to result in time-dependent expression. The expression from the promoter of the ribosomal protein-encoding gene RP30 (pRP30) was expected to be very strong during the early growth phase and highly downregulated during the lipid production phase. The expression of pGAPDH was expected to be high during the whole growth phase and only slightly downregulated during lipid production (Fig. 3A). The native OLE1 promoter was expected to be downregulated during the growth phase and upregulated during lipid production. We initially decided to exchange the native promoter of *OLE1* for pRP30 or pGAPDH, in strains expressing either *RtOLE1* or *AaOLE1* with and without FAD2-deletion (promoters were arbitrarily defined as 1000 bp upstream of the start codon of the respective genes).

All strains expressing the Ole1p version after pRP30 showed a substantial decrease in growth and lipid content (Fig. 3B), which could be recovered by supplementing the media with unsaturated fatty acids (Fig. 3C; Additional file 1: Fig. S4). The expression of *OLE1*-homologs with pRP30 is not sufficient to sustain a normal lipid production phase.

In contrast to pRP30, the pGAPDH-regulated strains showed similar undesired fatty acid profiles and growth as with the native *OLE1* promoter (cf. OKYL061 and OKYL063, Fig. 3B, OKYL056 and OKYL059, Fig. 2). Combining pGAPDH-regulated *OLE1* expression with the absence of *FAD2* also yielded lower growth and lipid content, albeit not as severe as observed when under the control of pRP30 (cf. KSYL09 and KSYL12, Fig. 3, KSYL15 and KSYL16 Fig. 2).

From our first experiments, the strains OKYL056 (pOLE1—*RtOLE1*) and OKYL061 (pGAPDH—*RtOLE1*) were the most promising cocoa butter production strains because of their good growth performances and desirable fatty acid profiles. Since deleting *FAD2* had a negative impact on the growth and lipid production when combined with promoter exchange of *RtOLE1*, we decided to continue with OKYL056 for our following experiments.

### Promoter exchange of *FAD2*

Our first experiments focused only on the Δ9 desaturase Ole1p, and we observed a negative impact on growth and lipid production if the Δ12 desaturase Fad2p was deleted in combination with an exchange of Ole1p or its promoter (Figs. 2, 3). To further understand the importance of Fad2p, we exchanged the native *FAD2* promoter for testing a range of expression levels. Primarily, we aimed to reduce the linoleic acid (C18:2) content, but we additionally tested strong promoters to explore the range of possible fatty acid profiles which might be of interest for other applications. The native OLE1 promoter is downregulated in the growth phase and upregulated in the lipid production phase. ICL1, RP30 and TEF1 promoter were expected to be very active during the growth phase and downregulated in the lipid production phase. Whereas pICL1 has a lower expression difference between growth and lipid phase. pGAPDH was reported to be active longer during growth (compared to pICL1, pRP30 and pTEF1) but still downregulated during lipid production (pOLE1—Cluster 5c; pICL1—Cluster 1b; pRP30 and pTEF1—Cluster 1a; pGAPDH—Cluster 3 [24]). We first conducted this experiment with strains expressing *RtOLE1*. *FAD2* expression under pICL1 yielded similar results as the native promoter, while pTEF1 and pOLE1 regulated FAD2 expression yielded similar results as with pGAPDH. Therefore, for the strain expressing native *YlOLE1* we discarded the pICL1, pTEF1 and pOLE1 regulated FAD2.

**Fig. 4:**
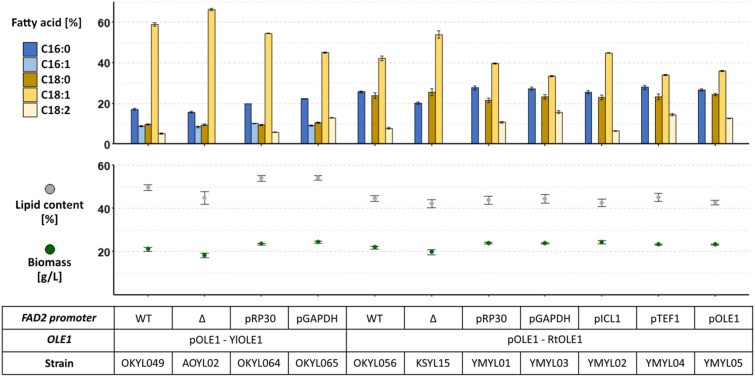
Promoter exchange of native *FAD2* gene. Strains were expressing the native *FAD2* gene under the control of different promoters. The strains were either expressing the native YlOLE1 or RtOLE1 under the control of the native OLE1 promoter, respectively. Strains were cultivated for 96 h in LPU media, and the fatty acid profile was determined by FAME extraction. Lipid content represents g FAME per g cell dry weight. Displayed is the mean and standard error of n ≥ 4 replicates

We found that the linoleic acid (C18:2) content in the fatty acid profile can vary without a substantial influence on the growth or lipid content (Fig. 4), ranging from no C18:2 (KSYL015) to 16% C18:2 (YMYL03). Additionally, oleic acid (C18:1) and linoleic acid (C18:2) were linked in an inverse correlated manner—similar to stearic acid (C18:0) and oleic acid (C18:1) in our first experiment (Fig. 3). In this case, however, the stearic acid (C18:0) content was largely unaffected by the changes of oleic and linoleic acid. We observed this correlation for both the native *OLE1* and *RtOLE1*.

Interestingly, the expression with the strong *TEF1* promoter [25] (YMYL04) resulted in a similar fatty acid profile as the expression with the *GAPDH* or *OLE1* promoter (YMYL03 and YMYL05) (Fig. 4), suggesting that high expression levels of desaturases are not the sole factor to influence the fatty acid profile, but that additional regulation exists.

Based on the fatty acid profile, we concluded that the strain OKYL056 (*FAD2* wt, *RtOLE1*) was the most promising strain for CBE production. The strain KSYL15 (fad2Δ, *RtOLE1*) also showed a promising fatty acid profile, however, the palmitic acid (C16:0) and oleic acid (C18:1) content of OKYL056 was closer to that of cocoa butter.

### Characterization of the most promising cocoa butter strain and related strains

To better understand and characterize the most promising CBE production strain (OKYL056), we monitored its growth, lipid production, and fatty acid profile over time in nitrogen-limited cultures. We also included the parental strain (OKYL049) and a strain with additional *FAD2* deletion (KSYL15) to understand the effect of the individual genetic modifications.

**Fig. 5:**
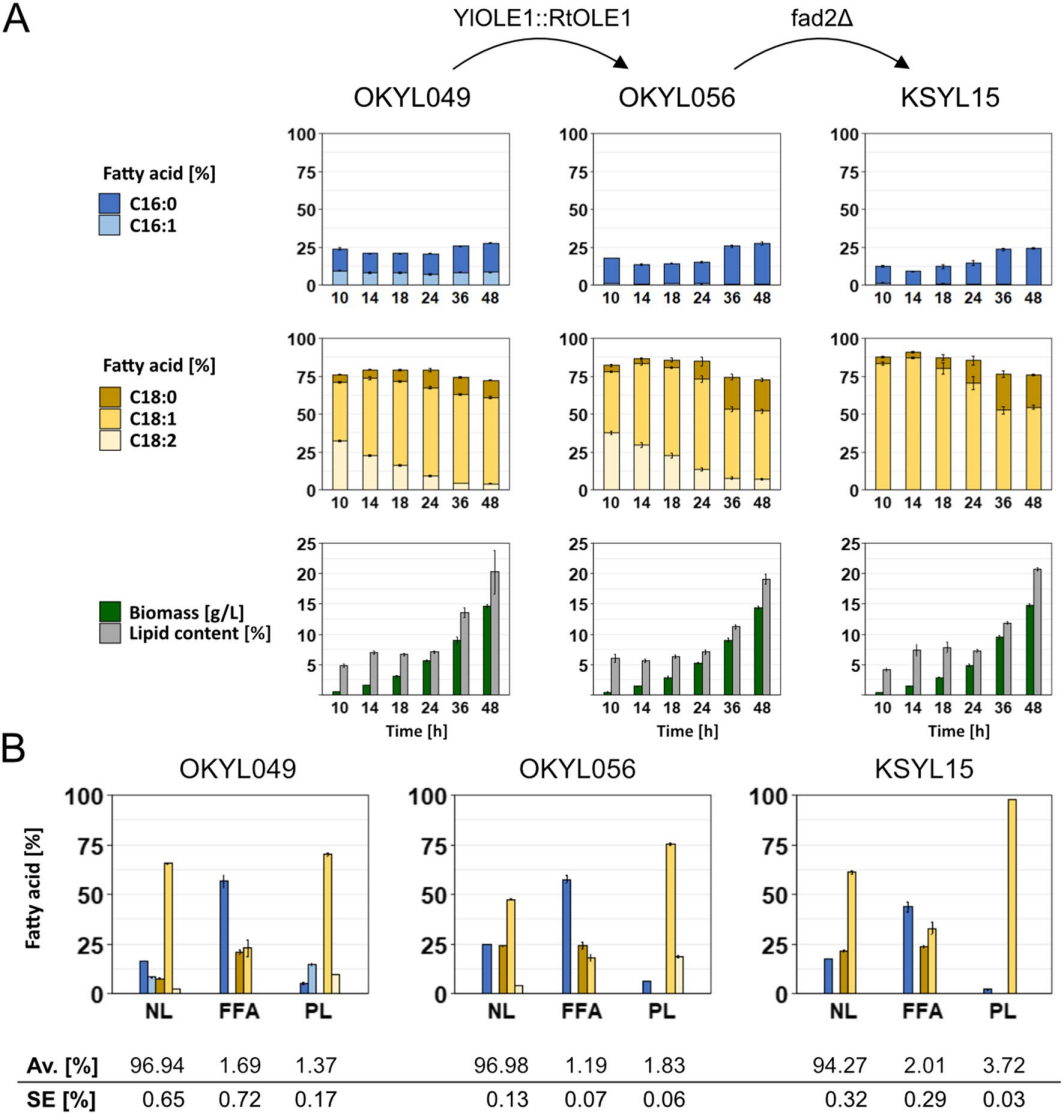
Fatty acid profile during growth and lipid class composition after 96 h of cultivation. Strains were cultivated in LPU media for up to 96 h. Displayed is the mean and standard error of n ≥ 4 replicates. **A** The fatty acid profile was determined by FAME extraction. Lipid content represents g FAME per g cell dry weight. **B** Cells were cultured for 96 h, and solid-phase extraction was performed, followed by FAME extraction of each fraction: neutral lipids (NL—including cholesterol, cholesterol ester, triacylglycerol, diacylglycerol, monoacylglycerol), free fatty acids (FFA) and phospholipids (PL). Bars show the percentage of the individual fatty acid of the total fatty acids from each fraction. The colour of the bars is the same as in **A**. The table shows the contribution of each lipid fraction to the sum of all fractions. *Av.* average, *SE* standard error

During the first 24 h after inoculation, lipid content remained stable while fatty acid profiles changed. We observed a shift to higher saturation in the C18 fatty acid species over time in all strains, while the ratio of C16 to C18 remained similar (~ 1:3). In the strains OKYL049 and OKYL056, the relative linoleic acid content (C18:2) decreased between 10 h (30%) and 24 h (10%). Simultaneously, the relative oleic acid content (C18:1) increased by approximately the same percentage (Fig. 5A). Except for the absence of palmitoleic acid (C16:1) in OKYL056, the fatty acid profiles of OKYL049 and OKYL056 were very similar during the first 24 h, while we previously observed that fatty acid profiles at 96 h were different (Fig. 2).

The *FAD2* deleted strain (KSYL15) lacks the Δ12 desaturase to produce linoleic acid (C18:2). Here the oleic acid content (C18:1) stayed constant over the first 18 h, and the amount of C18 fatty acids was similar to the other two strains despite the lack of linoleic acid (C18:2).

After 24 h, all strains started to accumulate lipids, and their fatty acid profiles changed between 24 and 36 h. Although the lipid content kept increasing after 36 h, the fatty acid profile remained constant (Fig. 5A).

These results showed that the fatty acid profile varied depending on the growth phase of the cultures. During the exponential phase, the change was mainly visible in the ratio of saturated to unsaturated fatty acids, while with the beginning of lipid accumulation, the shift occurred in the fatty acid chain length.

We performed solid-phase extraction (SPE) to further understand the growth limitations and the distribution of fatty acids in different lipid classes. We separated the neutral lipids (NL) that mainly consist of TAGs, free fatty acids (FFA) and phospholipids (PL) and analyzed the fatty acid distribution of each fraction.

The fatty acid composition of the neutral lipid (NL) fraction was very close to that of the total FAME fatty acid composition (Figs. 4, 5B) which most likely reflects the dominance of the NL (> 90% of total lipid). The composition of the free fatty acid pool was very different from the NL fraction, being devoid of palmitoleic acid (C16:1) and linoleic acid (C18:2). The absence of C18:2 can be explained by the functionality of the ∆12 desaturase, which only acts on fatty acids bound to membranes [15]. The absence of C16:1 in FFA is potentially of interest, but elucidating the reasons for this would require substantial further work. The phospholipid (PL) fatty acid composition was, in its turn, different from that of the NL and free fatty acids. We observed all dominant fatty acid species except for stearic acid (C18:0) in the PL fraction of the strain expressing both native desaturases (OKYL049). Oleic acid (C18:1) was the most dominant fatty acid with almost 75%. The oleic acid (C18:1) content increased to nearly 98% in KSYL15 (*RtOLE1* expression and *FAD2* deletion), and PL content increased from 1.83% (OKYL056) to 3.72% (KSYL15) with the deletion of *FAD2* (Fig. 5B). This result indicated that a higher amount of PL could compensate for the lack of polyunsaturated fatty acids (C18:2) in the PL fraction. Interestingly, overexpression of *FAD2* (YMYL03-05, Fig. 4) led to an overall increase of linoleic acid (C18:2) but did not decrease the total PL amount (Additional file 1: Fig. S5) compared to the wild type expression of *FAD2*. Different FAD2 expression levels increased both linoleic acid (C18:2) and palmitic acid (C16:0) in the PL (Additional file 1: Fig. S5), indicating a regulation of the cell to maintain membrane fluidity [26].

Additionally, the dominance of oleic acid (C18:1) in the PL fraction can explain why growth-deficient strains did show a higher recovery of growth when supplemented with oleic acid (C18:1) compared to palmitoleic acid (C16:1) (Fig. 2B; Additional file 1: Fig. S3).

## Discussion

This study shows that the fatty acid profile of *Y. lipolytica* can be altered to mimic cocoa butter by exchanging the Δ9 desaturase Ole1p. We found that growth and lipid production are depending on the expression of both desaturases, Ole1p and Fad2p. Taking this into account, the most promising strain for mimicking cocoa butter, in terms of fatty acid profile, growth, and lipid production, was obtained by exchanging the native *YlOLE1* with *OLE1* from *R.* *toruloides* (*RtOLE1*, strain OKYL056).

Our results showed a broader range of expression levels for *FAD2* without a negative impact on lipid production or growth than *OLE1*. For *FAD2*, high expression (TEF1 promoter), low expression (ICL1 promoter) and deletion yielded strains with high lipid content and different fatty acid profiles (Fig. 4). However, the expression of *OLE1* with different promoters yielded some strains with reduced growth and lipid production or complete growth deficiency (e.g. RP30 promoter) (Fig. 3). In fact, some of the most promising fatty acid profiles correlate with poor growth, e.g. *GtOLE1*, and *OLE1* homologs under control of the RP30 promoter. Adaptive laboratory evolution (ALE) experiments or targetted mutagenesis are approaches that could be used to further increase the growth and lipid content of such strains with favourable fatty acid profiles.

We found a link between the C18 fatty acids, which might be bottlenecks for future engineering of *Y. lipolytica* for tailored fatty acid profiles. When exchanging the native *OLE1* with other homologs, we observed that stearic and oleic acid (C18:0 and C18:1) are inversely correlated, while linoleic acid (C18:2) remained relatively unaffected (Fig. 2). During the promoter exchange of *FAD2,* we saw an inverse correlation between oleic and linoleic acid (C18:1 and C18:2), while stearic acid (C18:0) was unaffected (Fig. 4). However, these observations are based on total lipid extraction analysis. Because of the high contribution of the neutral lipid fraction (mainly TAG) to the total lipids (Fig. 5), a total lipid extraction is sufficient to estimate the TAG composition of strains. Therefore, the observed inverse correlation applies to TAGs but not necessarily to other lipid classes.

The separation of lipid classes with SPE allowed us to analyze the phospholipids (PL), the main building blocks of cell membranes. Within the PL, an increase of linoleic acid (C18:2) is accompanied by an increase of palmitic acid (C16:0) (Fig. 5; Additional file 1: Fig. S5). Polyunsaturated fatty acids (e.g. C18:2) increase the fluidity of biological membranes, while unsaturated fatty acids (e.g. C16:0) reduce their fluidity [26]. The cell might increase saturated fatty acids (C16:0) to maintain the membrane fluidity for optimal growth. Additionally, we found that oleic acid (C18:1) is the dominant fatty acid in the PL fraction, confirming previous findings [27]. In our engineered strain (KSYL15), oleic acid (C18:1) can make up 98% of the PLs without affecting growth or lipid production. We saw an increase in the total PL fraction in that strain, which could be used for metabolic engineering applications that use PLs as a precursor, e.g. branched fatty acids production [28].

In this study, we mainly focused on mimicking the fatty acid profile of cocoa butter. However, to create proper cocoa butter equivalents (CBE), the TAG structure of natural cocoa butter has to be imitated to ensure similar physical and chemical behaviour of the product [29]. A similar TAG structure can be achieved by introducing GPAT, LPAT and DGAT genes from *T. cacao,* as previously demonstrated in *S. cerevisiae* [6]. Most relevant for *Y. lipolytica* will be the Sn3 position (introduced by DGAT and PDAT), which in *Y. lipolytica* is naturally occupied by a monounsaturated fatty acid (C16:1 or C18:1), but in cocoa butter is occupied by a saturated fatty acid (C16:0 or C18:0). The Sn1 position in Y. lipolytica is mainly occupied by a saturated and Sn2 by an unsaturated fatty acid similar to cocoa butter. [19]

Other studies in *Y. lipolytica* to mimic cocoa butter have focused on the growth and fatty acid profile of wild-type strains when fed with different lipidic substrates (e.g. stearin, hydrolyzed oleic rapeseed oil, saturated free fatty acids) [30, 31]. Combining optimized media composition with genetically engineered strains can potentially allow for better tailored fatty acid profiles. This approach would also have the advantage of exploiting *Y. lipolytica*'s ability to consume a wide range of substrates (e.g. lipids or glycerol) to use waste streams as a low-cost carbon source [32, 33].

This study does yield promising results concerning CBE production and demonstrates the possibility of creating a pallet of strains expressing a wide range of fatty acid profiles while still maintaining a high lipid content that can be used for other applications apart from food/feed supplementation [34], e.g. biodiesel with different melting temperatures [35] or biopolymers [36].

## Conclusion

In this study, we used genetic engineering of the desaturases in *Y. lipolytica* and demonstrated the feasibility of producing fatty acid profiles to mimic cocoa butter and other oils. Our study demonstrates the wide range of fatty acid profiles that *Y. lipolytica* can produce while maintaining good growth and high lipid production. In combination with further genetic engineering and process optimization, e.g., alternative carbon sources, our results can contribute to establishing microbial cell factories for the sustainable production of food oils.

## Method

### Strains and strain construction

All *Yarrowia lipolytica* strains in this study are derived from the W29 background strain (Y-63746 from the ARS Culture Collection, Peoria, USA; a.k.a. ATCC20460/CBS7504). The initial starting strain of this study was OKYL029 which carries the *MHY1* deletion to prevent filamentous growth [20].

The obese strain (OKYL049) was constructed by additionally deleting the gene *ARE1* and overexpressing *DGA1* (Tef1in promoter, PEX20 terminator) [21] and is referred to as the wild type (WT) in this study. The genotype and parental relations of all strains are summarized in the Additional file 1: Table.

For the construction of gRNA plasmids for the CRISPR-Cas9 driven gene deletion and marker-free integration, the EasycloneYALI toolbox was used [37]. The deletion repair fragments were constructed from equal amounts of two single-stranded oligonucleotides (around 100 bp; 100 pmol/µL), incubated for 5 min at 95 °C and allowed to cool down to room temperature. 3 µL of this repair fragment was used for deletion. Integration cassettes for marker-free integration were obtained by digesting the corresponding plasmids (see Additional file 1). After a gel cleanup, 1 µg of repair fragment was used for transformation. For each transformation 500 ng of gRNA plasmid was used.

Transformation of *Y. lipolytica* was performed using a lithium-acetate based heat shock method previously described [38].

### Media and growth conditions

Lipid production media (LPU) consisted of 1.5 g/L yeast extract (Merck), 0.85 g/L, casamino acids (Formedium), 1.7 g/L Yeast Nitrogen Base without amino acids and ammonium sulfate (Formedium), 5.1 g/L potassium hydrogen phthalate (Merck) buffer adjusted to pH 5.5, 100 g/L glucose (Sigma-Aldrich), and 0.5 g/L urea (Sigma-Aldrich) [22]. For fatty acid supplementation, 1% (v/v) of Tween 20 (Merck) was added together with the indicated amount of fatty acid (palmitoleic, oleic, heptadecenoic acid, Sigma-Aldrich).

YPD plates contained 20 g/L peptone from meat (Merck), 10 g/L yeast extract, 20 g/L glucose, and 20 g/L agar (VWR). For selection, YPD plates were supplemented with 250 mg/L Nourseothricin (Jena Bioscience). LB plates contained 10 g/L peptone from casein (Merck), 10 g/L NaCl (Merck), 5 g/L yeast extract, 16 g/L agar and were set to pH 7.0 with 5 M NaOH (Merck).

All *Y. lipolytica* strains were cultivated in suspension at 30 °C in 10 mL media in 100 mL Erlenmeyer flasks at 200 rpm shaking unless differently stated.

*Escherichia coli* DH5 alpha was used for plasmid construction and purification and was cultivated in LB broth or on agar plates supplemented with 100 µg/mL ampicillin at 37 °C.

### Lipid extraction and quantification

*Y. lipolytica* strains were cultivated in LPU media for 96 h before fatty acid methyl ester (FAME) extraction to measure cellular lipid content. The protocol used was previously described [39]. In short, 100 µL of cell culture was spun down, the supernatant was discarded, and the cells were washed with 1 mL water. The suspension was spun down again, and the supernatant was removed. The cell pellet was dried in a vacuum dry freezer for one day. Then glyceryl triheptadecanoate (TAG(17:0/17:0/17:0)) (Sigma-Aldrich) was added to the cell pellet as an internal standard. 500 µL of a methanol solution containing 1 M NaOH was added, and the samples were vortexed at 1200 rpm at room temperature for 1 h. The solution was neutralized by carefully adding 80 µL of 50% sulfuric acid (Merck). The FAMEs were extracted by adding 500 µL *n*-hexane (Merck). Phases were separated by centrifugation for 1 min at 10,000×*g*. 200 µL of the upper hexane phase was mixed with 800 µL hexane, and 1 µL of this sample was analyzed on GC–MS (Thermo Scientific Trace 1310 coupled to a Thermo Scientific ISQ LT with a ZBFAME column (Phenomenex, length: 20 m; Inner Diameter: 0.18 mm; Film Thickness: 0.15 um)) with a method concisting of 2 min hold at 80 °C, followed by a ramp of 40 °C/min until 160 °C, a ramp of 5 °C/min until 185 °C, a ramp of 40 °C/min until 260 and a final hold of 260 °C for 30 s. The GLC reference standard GLC 403 was used as a standard curve.

The biomass/cell dry weight of each culture was calculated by filtration and allowed the calculation of lipid content as g FAME per g CDW [20].

### Solid-phase extraction (SPE)

Cell pellets of 100 µL culture were washed with 1 mL water and freeze-dried for one day. A mix of internal standards for each lipid fraction (Glyceryl triheptadecanoate, Pentadecanoic acid, 19:0 PC, Sigma-Aldrich) and ca. 50 µL of acid-washed glass beads were added. 1 ml of chloroform:methanol 2:1 (v:v) (Merck) was added, and the sample was vortexed at 1200 rpm at room temperature for 1 h. SPE cartridges (Supelclean™ LC-NH2 SPE Tube, Sigma-Aldrich) were placed on Vac Elut apparatus, activated by adding 4 mL of hexane, and Samples were loaded on the cartridges. Eluation of the different lipid fractions was achieved by adding 4 mL of chloroform:isopropanol 2:1 (v:v)(Merck) (neutral lipid fraction, including cholesterol, cholesterol ester, triacylglycerol (TAG), diacylglycerol (DAG), monoacylglycerol (MAG)), 2% acetic acid (Sigma-Aldrich) in diethyl ether (Sigma-Aldrich) (fatty acid fraction) and methanol (phospholipid fraction). The solvent of collected lipid fractions was evaporated and resuspended in 1 mL chloroform:methanol 2:1 (v:v), transferred to 1.5 mL reaction tubes and subject to FAME extraction before analysis on GC–MS. [40] For SPE samples the GLC reference standard GLC 426 was used, and the method concisting of 2 min hold at 80 °C, followed by a ramp of 40 °C/min until 120 °C, a ramp of 3.5 °C/min until 165 °C, a ramp of 40 °C/min until 260 and a final hold of 260 °C for 30 s.

### Growth profiler

*Y. lipolytica* strains were grown in LPU media overnight and inoculated in 150 µL of LPU media to a starting OD_600_ of 0.05 in 96-well plates. The cells were cultivated in quadruplicates at 30 °C and 200 rpm. The growth was measured every 30 min by the Growth Profiler 960 (Enzyscreen B.V., Heemstede, The Netherlands).

## Supplementary Information

**Additional file 1.** Supplementary figures and list of Strains, Primers, and DNA sequences used in this study.

**Additional file 2.** Raw data.

## Data Availability

The datasets supporting the conclusions of this article are included within the article and its Additional files.

## Abbreviations

C16:0: Palmitic acid
C16:1: Palmitoleic acid
C18:0: Stearic acid
C18:1: Oleic acid
C18:2: Linoleic acid
ALE: Adaptive laboratory evolution
CBE: Cocoa butter equivalents
DGA: Diacylglycerol
FAME: Fatty acid methyl ester
FFA: Free fatty acids
GC–MS: Gas chromatography–mass spectrometry
HPLC: High-performance liquid chromatography
MAG: Monoacylglycerol
NL: Neutral lipids
ORF: Open reading frame
PL: Phospholipids
SPE: Solid-phase extraction
TAG: Triacylglyceride
