# Supplementary material for: Altering the fatty acid profile of Yarrowia lipolytica to mimic cocoa butter by genetic engineering of desaturases
Source: Microb Cell Fact. 2022 Feb 19;21:25. doi: 10.1186/s12934-022-01748-x (PMC8857786; doi:10.1186/s12934-022-01748-x)
Supplement: Supplementary file 1 — Additional file 1. Supplementary figures and list of Strains, Primers, and DNA sequences used in this study. [file 12934_2022_1748_MOESM1_ESM.pdf]

Supplementary Konzock et al 2022

<u>Content</u>

Figure S1: Deletion of OLE1

Figure S2: Fatty acid profile and growth performance of additional strains

Figure S3: Growth profiler growth curves with supplementation of unsaturated fatty acids.

Figure S4: Growth profiler growth curves of all strains sorted by figures of main text.

Figure S5: SPE data of Fad2 deletion strains

Supplementary list 1: List of Yarrowia lipolytica strains

Supplementary list 2: List of plasmids

Supplementary list 3: List of primers

Supplementary list 4: List of genetic elements

Supplementary figures

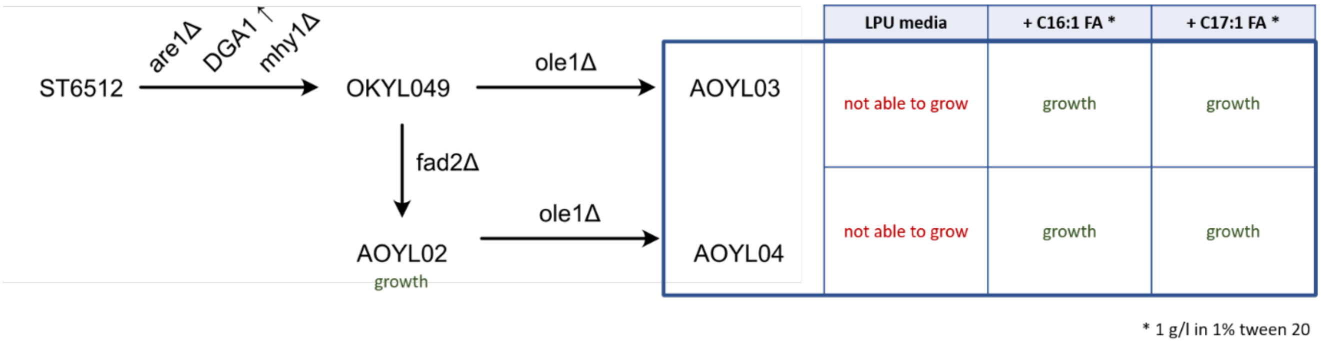

Figure S1: Deletion of OLE1 is lethal but can be recovered by supplementation of unsaturated fatty acids. Strains with OLE1 deletion are not viable unless unsaturated fatty acid was supplemented. Transformation of strains was only possible if unsaturated fatty acids were supplemented to the YPD NAT plates. Interestingly, supplementation of uneven-numbered unsaturated fatty acids also recovered growth. Deletion of the Δ12 desaturase FAD2 alone did not cause growth defects.

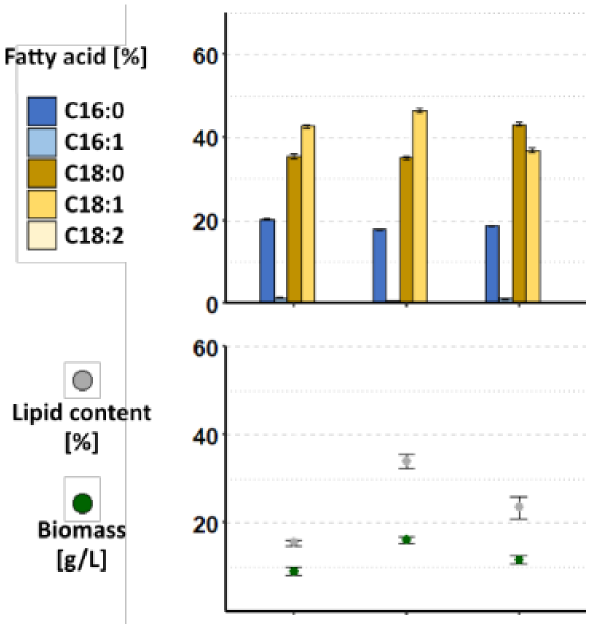

FAD2	Δ		
OLE1	Gt	Rt	Aa
Promoter	pGAPDH	pICL1	
Strain	KSYL06	KSYL08	KSYL11

Figure S2 Fatty acid profile and growth performance of additional strains. The native OLE1 gene was exchanged for either RtOLE1 or AaOLE1 and the native promoter was exchanged for either pGAPDH or pICL1. Strains were cultivated for 96 h in LPU media and the fatty acid profile was determined by FAME extraction. Lipid content represents g FAME per g cell dry weight. Displayed is the mean and standard error of n ≥ 4 replicates.

The fatty acid profiles of the strains expressing the *OLE1* homologs under the control of pICL (KSYL08 and KSYL11) were very similar to that of the strain expressing the *OLE1* homologs under the control of the native *OLE1* promoter (KSYL15 and KSYL16, **Figure 2**). We chose to continue with pOLE1-*RtOLE1,* because of its good growth and lipid production. Additional we wanted to introduce as few genetic changes as possible to keep the native lipid regulation intact.

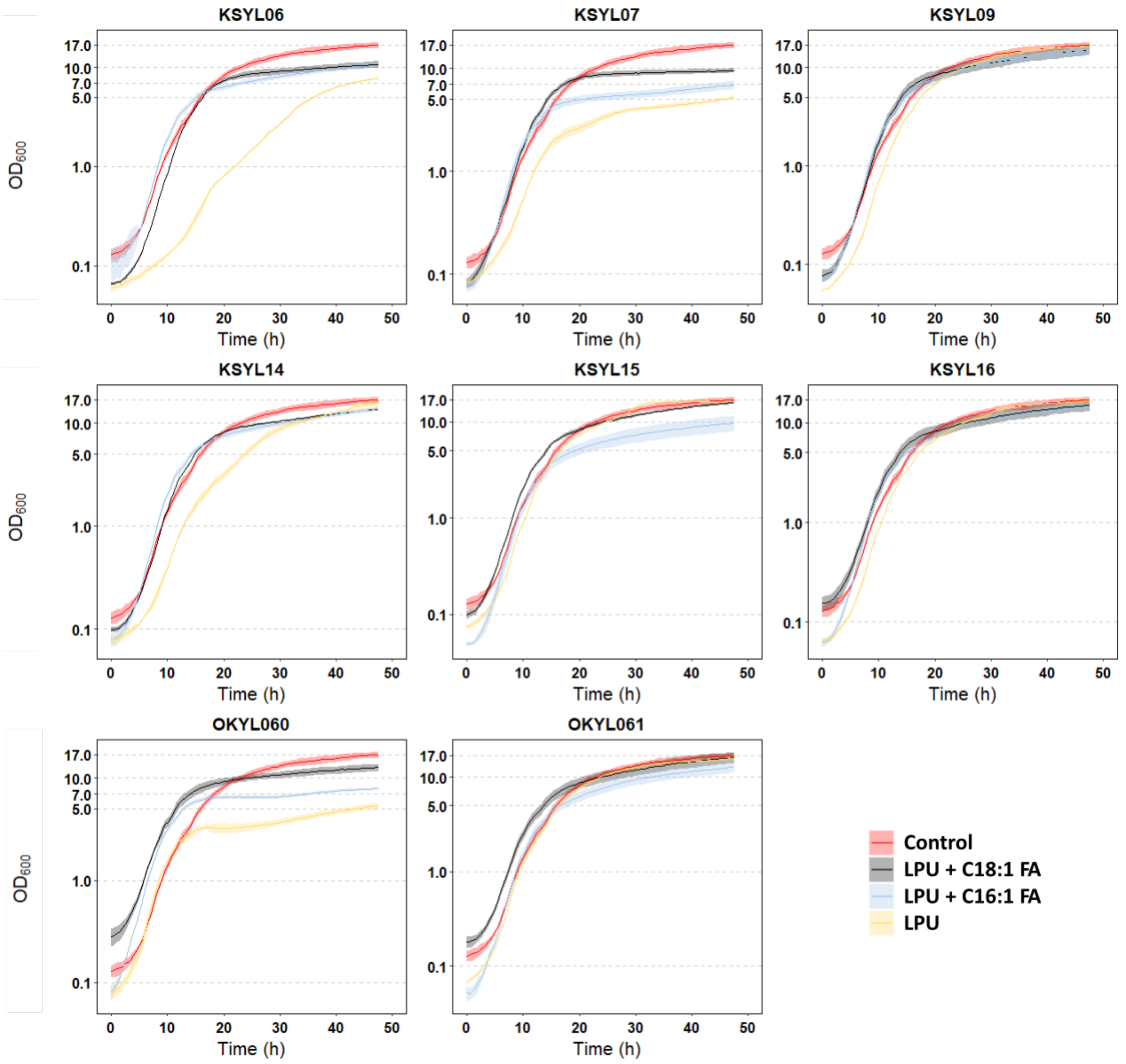

Figure S3: Growth profiler growth curves with supplementation of unsaturated fatty acids. Cells were cultivated in 96-well plates in LPU media with or without the addition of palmitoleic acid (C16:1) or oleic acid (C18:1) (500 mg/L in 1% tween 20) and OD_{600} was measured every 30 min. The lines and shadows represent the average and standard deviation of quadruplicates, respectively. Control: OKYL049 in LPU media without FA supplementation.

OKYL049 is the wild type strain that was engineered for high lipid production but with no modifications of OLE1 or FAD2. Strains that showed a reduced growth could recover their growth by fatty acid supplementation. The same supplementation did not further increase the growth of healthy strains (e.g. KSYL09).

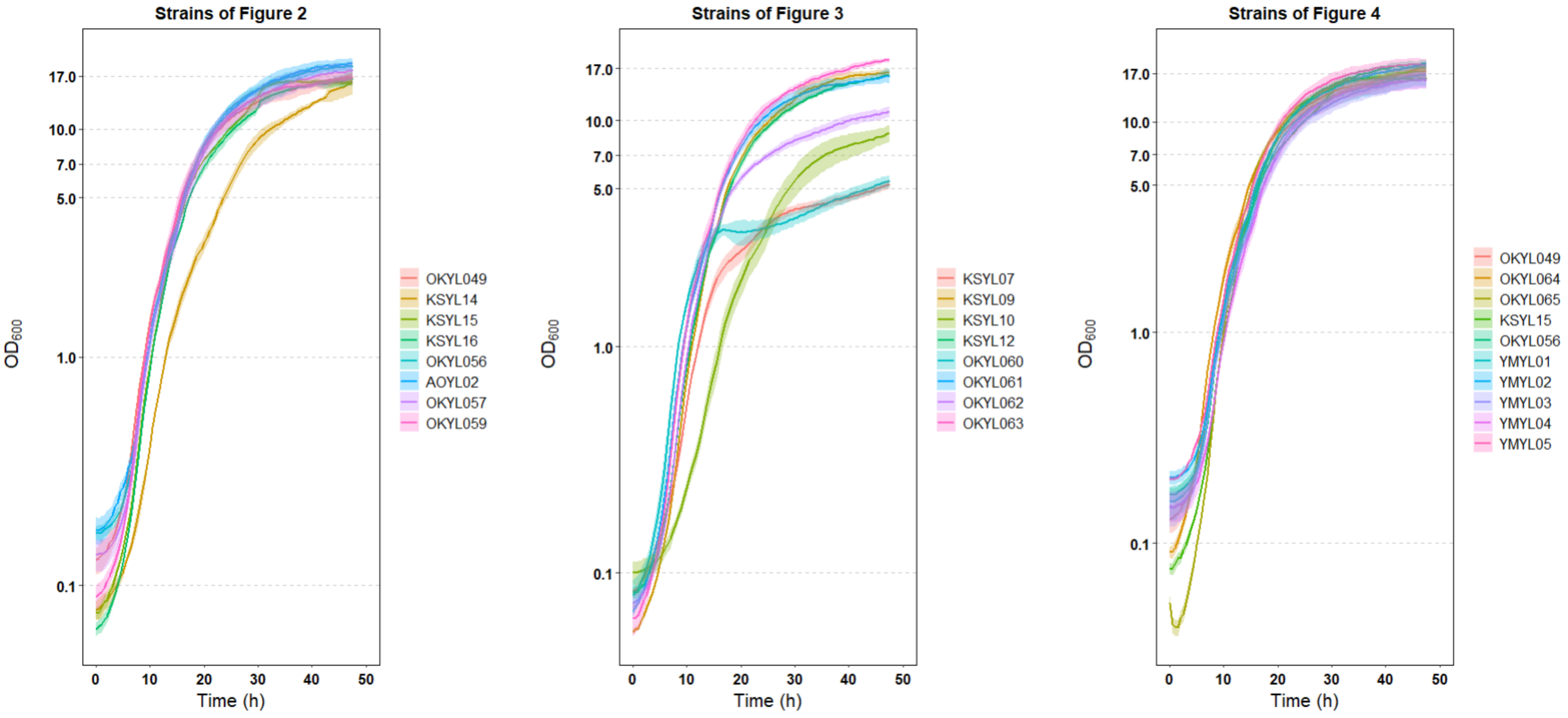

Figure S4: Growth profiler growth curves of all strains sorted by figures of main text. Cells were cultivated in 96-well plates in LPU media and OD_{600} was measured every 30 min. The lines and shadows represent the average and standard deviation of quadruplicates, respectively.

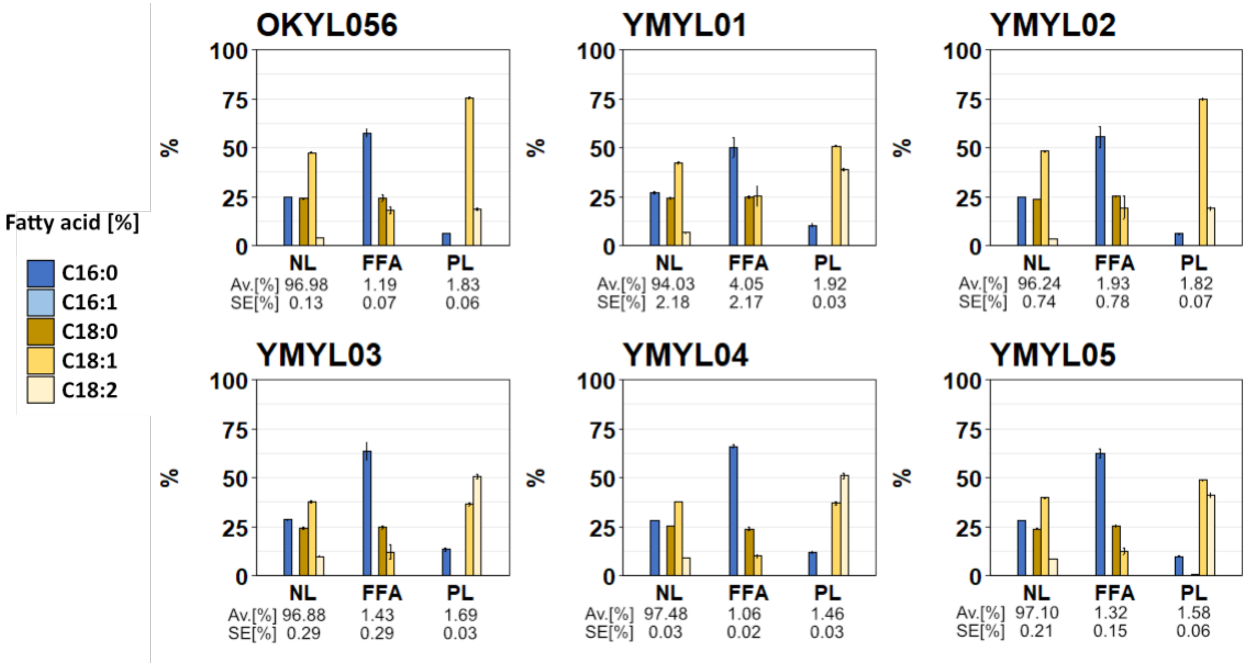

Figure S5: SPE data of Fad2 deletion strains. Displayed is the mean and standard error of n = 4 replicates. Cells were cultured in LPU media for 96 h and solid-phase extraction was performed, followed by FAME extraction of each fraction: neutral lipids (NL – including cholesterol, cholesterol ester, triacylglycerol, diacylglycerol, monoacylglycerol), free fatty acids (FFA) and phospholipids (PL). The table shows the contribution of each lipid fraction to the sum of all fractions. Av. = average, SE = standard error.

Supplementary tables

Supplementary list 1: List of *Yarrowia lipolytica* strains used in the study - Konzock et al 2022

Visiualization of strain relations

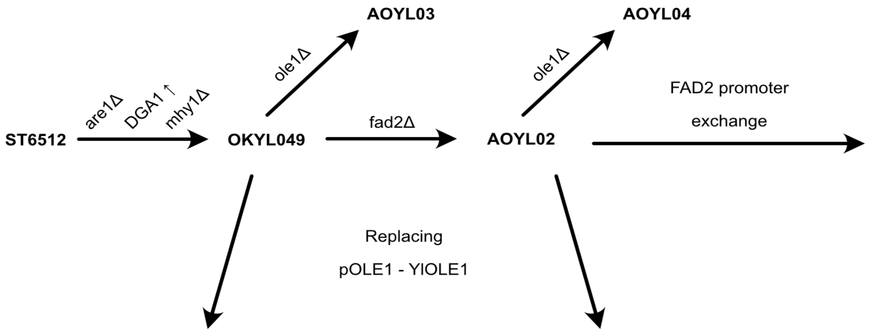

FAD2 promoter	
pRP30	pGAPDH
OKYL064	OKYL065

promoter	OLE1 homolog: GtOLE1	RtOLE1	AaOLE1
pRP30		OKYL060	OKYL062
pGAPDH		OKYL061	OKYL063
pOLE1	OKYL057	OKYL056	OKYL059

promoter	OLE1 homolog: GtOLE1	RtOLE1	AaOLE1
pRP30		KSYL07	KSYL10
pICL		KSYL08	KSYL11
pGAPDH	KSYL06	KSYL09	KSYL12
pOLE1	KSYL14	<u>**KSYL15**</u>	KSYL16

Re-introduction of FAD2 with different promoters

FAD2 promoter				
pRP30	pICL1	pGAPDH	pTEF1	pOLE1
YLYM01	YLYM02	YLYM03	YLYM04	YLYM05

NAME	Genotype description	Reference
**ST6512 (wild type)**	MATa ku70Δ::Cas9+DsdA	Eko Roy Marella, et al., Metabolic Engineering, https://doi.org/10.1016/j.ymben.2019.08.009
**OKYL049 (*obese strain*)**	MATa ku70Δ::Cas9+DsdA E1::pTef1in+DGA1+tPEX20 are1Δ mhy1Δ	Konzock, O., Zaghen, S. & Norbeck, J. Tolerance of Yarrowia lipolytica to inhibitors commonly found in lignocellulosic hydrolysates. BMC Microbiol 21, 77 (2021). https://doi.org/10.1186/s12866-021-02126-0
**AOYL02**	MATa ku70Δ::Cas9+DsdA E1::pTef1in+DGA1+tPEX20 are1Δ mhy1Δ fad2Δ	this study
**AOYL03**	MATa ku70Δ::Cas9+DsdA E1::pTef1in+DGA1+tPEX20 are1Δ mhy1Δ ole1Δ	this study
**AOYL04**	MATa ku70Δ::Cas9+DsdA E1::pTef1in+DGA1+tPEX20 are1Δ mhy1Δ fad2Δ ole1Δ	this study
**OLE1 exchange in AOYL02**		
**KSYL04**	MATa ku70Δ::Cas9+DsdA E1::pTef1in+DGA1+tPEX20 are1Δ mhy1Δ fad2Δ pole1+ole1Δ::pRP30+GtOLE1	this study
**KSYL05**	MATa ku70Δ::Cas9+DsdA E1::pTef1in+DGA1+tPEX20 are1Δ mhy1Δ fad2Δ pole1+ole1Δ::pICL1+GtOLE1	this study
**KSYL06**	MATa ku70Δ::Cas9+DsdA E1::pTef1in+DGA1+tPEX20 are1Δ mhy1Δ fad2Δ pole1+ole1Δ::pGAPDH+GtOLE1	this study
**KSYL07**	MATa ku70Δ::Cas9+DsdA E1::pTef1in+DGA1+tPEX20 are1Δ mhy1Δ fad2Δ pole1+ole1Δ::pRP30+RtOLE1	this study
**KSYL08**	MATa ku70Δ::Cas9+DsdA E1::pTef1in+DGA1+tPEX20 are1Δ mhy1Δ fad2Δ pole1+ole1Δ::pICL1+RtOLE1	this study
**KSYL09**	MATa ku70Δ::Cas9+DsdA E1::pTef1in+DGA1+tPEX20 are1Δ mhy1Δ fad2Δ pole1+ole1Δ::pGAPDH+RtOLE1	this study
**KSYL10**	MATa ku70Δ::Cas9+DsdA E1::pTef1in+DGA1+tPEX20 are1Δ mhy1Δ fad2Δ pole1+ole1Δ::pRP30+AaOLE1	this study
**KSYL11**	MATa Δku70::Cas9::DsdA E1::pTef1in+DGA1+tPEX20 are1Δ mhy1Δ fad2Δ pole1+ole1Δ::pICL1+AaOLE1	this study
**KSYL12**	MATa ku70Δ::Cas9+DsdA E1::pTef1in+DGA1+tPEX20 are1Δ mhy1Δ fad2Δ pole1+ole1Δ::pGAPDH+AaOLE1	this study
**KSYL14**	MATa ku70Δ::Cas9+DsdA E1::pTef1in+DGA1+tPEX20 are1Δ mhy1Δ fad2Δ ole1Δ::GtOLE1	this study
**KSYL15**	MATa ku70Δ::Cas9+DsdA E1::pTef1in+DGA1+tPEX20 are1Δ mhy1Δ fad2Δ ole1Δ::RtOLE1	this study
**KSYL16**	MATa ku70Δ::Cas9+DsdA E1::pTef1in+DGA1+tPEX20 are1Δ mhy1Δ fad2Δ ole1Δ::AaOLE1	this study
**OLE1 exchange in OKYL049**		
**OKYL056**	MATa ku70Δ::Cas9+DsdA E1::pTef1in+DGA1+tPEX20 are1Δ mhy1Δ ole1Δ::RtOLE1	this study
**OKYL057**	MATa ku70Δ::Cas9+DsdA E1::pTef1in+DGA1+tPEX20 are1Δ mhy1Δ ole1Δ::GtOLE1	this study
**OKYL059**	MATa ku70Δ::Cas9+DsdA E1::pTef1in+DGA1+tPEX20 are1Δ mhy1Δ ole1Δ::AaOLE1	this study
**OKYL060**	MATa ku70Δ::Cas9+DsdA E1::pTef1in+DGA1+tPEX20 are1Δ mhy1Δ pole1+ole1Δ::pRP30+RtOLE1	this study
**OKYL061**	MATa ku70Δ::Cas9+DsdA E1::pTef1in+DGA1+tPEX20 are1Δ mhy1Δ pole1+ole1Δ::pGAPDH+RtOLE1	this study
**OKYL062**	MATa ku70Δ::Cas9+DsdA E1::pTef1in+DGA1+tPEX20 are1Δ mhy1Δ pole1+ole1Δ::pRP30+AaOLE1	this study
**OKYL063**	MATa ku70Δ::Cas9+DsdA E1::pTef1in+DGA1+tPEX20 are1Δ mhy1Δ pole1+ole1Δ::pGAPDH+AaOLE1	this study
**FAD2 promoter exchange in AOYL02**		
**OKYL064**	MATa ku70Δ::Cas9+DsdA E1::pTef1in+DGA1+tPEX20 are1Δ mhy1Δ pFAD2::pRP30	this study
**OKYL065**	MATa ku70Δ::Cas9+DsdA E1::pTef1in+DGA1+tPEX20 are1Δ mhy1Δ pFAD2::pGAPDH	this study

re-introduction of FAD2 with different promoters in KSYL15		
**YMYL01**	MATa ku70Δ::Cas9+DsdA E1::pTef1in+DGA1+tPEX20 are1Δ mhy1Δ fad2Δ::pRP30+FAD2 ole1Δ::RtOLE1	this study
**YMYL02**	MATa ku70Δ::Cas9+DsdA E1::pTef1in+DGA1+tPEX20 are1Δ mhy1Δ fad2Δ::pICL1+FAD2 ole1Δ::RtOLE1	this study
**YMYL03**	MATa ku70Δ::Cas9+DsdA E1::pTef1in+DGA1+tPEX20 are1Δ mhy1Δ fad2Δ::pGAPDH+FAD2 ole1Δ::RtOLE1	this study
**YMYL04**	MATa ku70Δ::Cas9+DsdA E1::pTef1in+DGA1+tPEX20 are1Δ mhy1Δ fad2Δ::pTEF1+FAD2 ole1Δ::RtOLE1	this study
**YMYL05**	MATa ku70Δ::Cas9+DsdA E1::pTef1in+DGA1+tPEX20 are1Δ mhy1Δ fad2Δ::pOLE1+FAD2 ole1Δ::RtOLE1	this study

Supplementary list 2: List of plasmids used in the study - Konzock et al 2022

NAME	Comments	*Y. lipolytica* marker	Purpose of plasmid
**OKEC048**	FAD2 2x cut cassette	Nourseothricin	gRNA plasmids for FAD2 locus (delta-12-desaturase)
**OKEC055**	OLE1 2x cut cassette (in promoter and ORF)	Nourseothricin	gRNA plasmid to knock out OLE1 with promoter to replace it with different OLE1 versions
**OKEC059**	pOLE1-OLE1::pRP30-GtOLE1	none	repair fragment plasmid for integration of diffeent OLE1 versions with different promoters into the OLE1 locus
**OKEC060**	pOLE1-OLE1::pICL1-GtOLE1	none	
**OKEC061**	pOLE1-OLE1::pGAPDH-GtOLE1	none	
**OKEC062**	pOLE1-OLE1::pRP30-RtOLE1	none	
**OKEC063**	pOLE1-OLE1::pICL1-RtOLE1	none	
**OKEC064**	pOLE1-OLE1::pGAPDH-RtOLE1	none	
**OKEC065**	pOLE1-OLE1::pRP30-AaOLE1	none	
**OKEC066**	pOLE1-OLE1::pICL1-AaOLE1	none	
**OKEC067**	pOLE1-OLE1::pGAPDH-AaOLE1	none	
**OKEC069**	OLE1::GtOLE1	none	repair fragment plasmids for 4 different OLE1 versions replacing the ORF of YlOLE1, without changing the native OLE1 promoter
**OKEC070**	OLE1::RtOLE1	none	
**OKEC071**	OLE1::AaOLE1	none	
**OKEC072**	OLE1 2x cut cassette (only in ORF)	Nourseothricin	gRNA plasmid to knock out OLE1 alone but leave the promoter
**OKEC083**	pFAD2-FAD2::pRP30-FAD2	none	repair fragment plasmid for integration of FAD2 with different promoters into the FAD2 locus
**OKEC084**	pFAD2-FAD2::pICL-FAD2	none	
**OKEC085**	pFAD2-FAD2::pGAPDH-FAD2	none	
**OKEC086**	pFAD2-FAD2::pOLE1-FAD2	none	
**OKEC087**	pFAD2-FAD2::pOLE1-FAD2	none	
**OKEC088**	pFAD2-FAD2::pKEX2-FAD2	none	

Supplementary list 3: List of primers used in the study - Konzock et al 2022

Name	Sequence	additional info
OLE1_cut-site_1_FW	tggaggttagaggttagagggttttagagct	gRNA plasmid for OLE1
OLE1_cut-site_1_RW	cctctaacctctaacctccataaccaacct	
OLE1_cut-site_2_FW	ggtaccgctgtcttcaacggggttttagagct	
OLE1_cut-site_2_RW	ccgttgaagacagcggtacctaaccaacct	
OLE1_cut-site_1.2_FW	gtagttgacatctcggccgggttttagagct	combine with OLE1 cut site 2 to form OKEC072
OLE1_cut-site_1.2_RW	ccggccgagatgtcaactactaaccaacct	
basic_vector_BB_RV	CATTCGCGGCCGCAAATTTA	primer to amplify fragment for the construction of the repair fragment plasmids to exchange OLE1 with different promoters
basic_vector_BB_FW	CATTCGCGGCCGCATTTAAATCC	
basic-vector_Screen_FW	CGACTCACTATAGAAGTTCC	
basic-vector_Screen_RV	CGACTCTATAGTGAAGTTCC	
OLE1_up_500_FW	TCATTTTATTTAAATTTGCGGCCGCGAATGTCCTTTGGGCGGGAGTGATTGG	
OLE1_up_500_RV	GAATAAGTATCGTATGTACAGTAGATACAG	
OLE1_up_500_RV(short)	AGGAACTTCTGAAGTGGGGA	
GtOLE1_FW_(pRP30)	CAAGCCCGAACGACACACCCACACACAACCATGGCGACCTACACGCC	
Rt-OLE1_FW_(pRP30)	CAAGCCCGAACGACACACCCACACACAACCATGACTGCCTCTTCGGCACTC	
Aa-OLE1_FW_(pRP30)	CAAGCCCGAACGACACACCCACACACAACCATGAACGGTCCCGAAGAGGTG	
GtOLE1_FW_(pICL1)	GGTGCCCTCGACCCCGTCCAGGTGACCCAGATGGCGACCTACACGCC	
Rt-OLE1_FW_(pICL1)	GGTGCCCTCGACCCCGTCCAGGTGACCCAGATGACTGCCTCTTCGGCACTC	
Aa-OLE1_FW_(pICL1)	GGTGCCCTCGACCCCGTCCAGGTGACCCAGATGAACGGTCCCGAAGAGGTG	
GtOLE1_FW_(pGAPDH)	ATTCATTCTTGAATTAAACACACATCAACAATGGCGACCTACACGCC	
Rt-OLE1_FW_(pGAPDH)	ATTCATTCTTGAATTAAACACACATCAACAATGACTGCCTCTTCGGCACTC	
Aa-OLE1_FW_(pGAPDH)	ATTCATTCTTGAATTAAACACACATCAACAATGAACGGTCCCGAAGAGGTG	
pRP30_FW_(OLE1_up)	CTGTATCTACTGTACATACGATACTTATTCCGAACTGTGTAGGAATGAAAC	
pRP30_RV	GGTTGTGTGTGGGTGTGTCG	
pICL1_FW_(OLE1_up)	CTGTATCTACTGTACATACGATACTTATTCGTAGCGTTGGTCTGTCCTGTCG	
pICL1_RV	CTGGGTCACCTGGACGGGGTC	
pGAPDH_FW_(OLE1_up)	CTGTATCTACTGTACATACGATACTTATTCGGTTGAAATGAATCGGCCGACG	
pGAPDH_RV	TGTTGATGTGTGTTTAATTCAAG	
OLE1_up_1500_RV	GTTGAATGTGTAGTTAGTAGATGAGG	
GtOLE1_FW_(pYlOLE1)	CTTTCCTCATCTACTAACTACACATTCAACATGGCGACCTACACGCCGCC	
Rt-OLE1_FW_(pYlOLE1)	CTTTCCTCATCTACTAACTACACATTCAACATGACTGCCTCTTCGGCACTCG	

**Aa-OLE1_FW_(pYlOLE1)**	CTTTCCTCATCTACTAACTACACATTCAACATGAACGGTCCCGAAGAGGTG	
**OLE1_up_up_screen_FW**	AACTGCAAACAACCGGGACA	for screening the integration of the different OLE1 genes and promoters into OLE1 locus. FW binds in gDNA, outside of RF, RV binds around 200 -500 bp after ATG of ORF.
**AaOLE1_screen_RV**	ACCGACAAAAAGCCGATTGC	
**RtOLE1_screen_RV**	CGAATACGGGTCGAGGTCTG	
**GtOLE1_screen_RV**	CCACCATTTGATGGAGCCCT	
**pFAD2upper_(backbone)_FW**	ATTTTATTTAAATTTGCGGCCGCGAATGagtccaactcggttagtccaacc	primer to amplify fragment for the construction of the repair fragment plasmids to introduce FAD2 with different promoters
**pFAD2upper_RV**	acaagcacggtcccattgtaacatgtg	
**tFAD_FW**	atggattcgaccacgcagacc	
**tFAD2_(backbone)_RV**	GAAGTGGGGATTTAAATGCGGCCGCGAATGacagtagactgctcgtacagtagtctctc	
**pRP30_FW**	agacacatgttacaatgggaccgtgcttgtcgaactgtgtaggaatgaaacaacaacatagac	
**pRP30_(tFAD2)_RV**	gccggtgttggtctgcgtggtcgaatccatggttgtgtgtgggtgtgtcgttcg	
**pICL1_(pFAD2upper)_FW**	agacacatgttacaatgggaccgtgcttgtgtagcgttggtctgtcctgtcg	
**pICL1_(FAD2)_RV**	gccggtgttggtctgcgtggtcgaatccatctgggtcacctggacggggtc	
**pGAPDH_(pFAD2upper)_FW**	agacacatgttacaatgggaccgtgcttgtggttgaaatgaatcggccgacgc	
**pGAPDH_(FAD2)_RV**	gccggtgttggtctgcgtggtcgaatccattgttgatgtgtgtttaattcaag	
**pOLE1_(pFAD2upper)_FW**	AGACACATGTTACAATGGGACCGTGCTTGTTTCTATTGTGGAACTCGTCTCTAC	
**pOLE1_(FAD2)_RV**	GCCGGTGTTGGTCTGCGTGGTCGAATCCATGTTGAATGTGTAGTTAGTAGATGAGG	
**pTEF1_(pFAD2upper)_FW**	AGACACATGTTACAATGGGACCGTGCTTGTAGAGACCGGGTTGGCGGCG	
**pTEF1_(FAD2)_RV**	GCCGGTGTTGGTCTGCGTGGTCGAATCCATTTTGAATGATTCTTATACTCAGAAGGAAATGC	
**pKEX2_(pFAD2upper)_FW**	AGACACATGTTACAATGGGACCGTGCTTGTACACACACAAGACACGCGTGC	
**pKEX2_(FAD2)_RV**	GCCGGTGTTGGTCTGCGTGGTCGAATCCATATCGTACCGTCGCTGATGTCTTTTTG	
**pFAD2-cut-site_1_FW**	caaaagctgagggtaaacaggttttagagct	gRNA plasmid for FAD2
**pFAD2-cut-site_1_RV**	ctgtttaccctcagcttttgtaaccaacct	
**pFAD2-cut-site_2_FW**	tcgtacacactcttgcacaagttttagagct	
**pFAD2-cut-site_2_RV**	ttgtgcaagagtgtgtacgataaccaacct	

marked in orange are the overhangs for the Easyclone USER cloning system

Supplementary list 4: List of genetic elements used in the study - Konzock et al 2022

	Name	YALI0	YALI1	Sequence
Promoter	pTEF1			AGAGACCGGGTTGGCGGCGTATTTGTGTCCCAAAAAACAGCCCCAATTGCCCCAATTGACCCCAAATTGACCCAGTAGCGGGCCCAACCCCGGCGAGAGCCCCCTTCACCCCACATATCAAACCTCCCCCGGTTCCCACACTTGCCGTTAAGGGCGTAGGGTACTGCAGTCTGGAATCTACGCTTGTTCAGACTTTGTACTAGTTTCTTTGTCTGGCCATCCGGGTAACCCATGCCGGACGCAAAATAGACTACTGAAAATTTTTTTGCTTTGTGGTTGGGACTTTAGCCAAGGGTATAAAAGACCACCGTCCCCGAATTACCTTTCCTCTTCTTTTCTCTCTCTCCTTGTCAACTCACACCCGAAATCGTTAAGCATTTCCTTCTGAGTATAAGAATCATTCAAA
	pICL1			gtagcgttggtctgtcctgtcgcactctgttcaaagacagaagaaagaaaaagctaacctccacgtcagagacaatggtagaaggcttgttccttgcaaccgaggagagtgagtgttctcggcacgagcatcatgggcgatctggagggtatttttgaggggaaaaaacgggatcaggacaaacagaggccacagaccgggaatctgggccccaaaacggccttttcccgtcgcaaaaccggtctacatacaccccttcggcccgccacaggccggtgtgaaaaaccctaaagcttgcttcaaaccagacggacgcacagcaagacacatcatgaagagtcacctgcagtatatatagatctggggatccccagtagactgaccaagcatacaaaaagtgagtatccaacagcgacacgtgagatggcagagacacagagacgtgtctacatggttggacaagtctccacattcgccagagacgtatccacatacaaacacaatctcacagctgatctgctcctgtgacagcacagtacatgttagtggatgaggtgttgtgtggtgggttaaatgggtggactgattcagtggcatcggtggcgacaccctctactcttcatgtcgtcacctaccgttcggaatcccaaaaatctgatgaactaaacgatttctggccaaaacacaattttgccaaagaagtcggtctcaccaatgcaagtgtcacatcaaacatctgtcccgtactaacccagtgtccgaacagcagcgattcaacaacgaagtcgaggagatcaagaagtggtggtcttccccccgatggaagcacaccaagcgtgtctactctcccgaggacattgcctcccgacgaggaaccatcaaggtcccccaggcctcttctcagcaggctgacaagctcttcaagctgctccaggagcacgagaagaaccacaccgcctccttcacctacggtgccctcgaccccgtccaggtgacccag
	pRP30			cgaactgtgtaggaatgaaacaacaacatagactcgctaatactgatctcacagtaacaaaagtagtcttattttgaatctggaatctcactgagtctttgaggcacttttactcccattggtcctcacatttcacgacttgtaaagacaaaagtttttctatcgtaacgcagggaagaagcgaccttagcgtccgatactaacatggacgaaatacttgtaaggatacttgcaacgtacttgtacttgtacaacccaaccctttccaattggatatgcgcaaacacagtggacatcattccaccagatgcggcaccagactcgtatcggcagcaccattgtcctcgtgacttttgcgctaacgaaacactatttggtctctaaccccacagtgtggagaagggccgacaggagggctcgagaagcgatctcgagtgaatctgactttgattggtgtggtattactggtcgatacgagtaagacgcgagcacttgaactcgcatccattcaatacagaagacgatctcatgagacacgtgtacaagtacaagtatgctgaatcagaggtcgcctaaagtatgtacttacccctcgtattgagccgtaaccattacccctcaggcggagattcgactctggattgattccagactagatcagactctagaaccaatccagatcagctccagatcagctccagatcagctcaaatcagctccatcacaacccttacaaacgtccacgatactcgttgagcaatatctgcagtcatgccataaagttcatcgatattgtagttgcacttttgtgcacttttgagagggtgtggaataaggttttgtttccccatttagtcgattttatcacatgatacagctagggttagccctaacgtcccaaacgggacaggaccacgcgcggagcctggcacttttgcaaagttactctggcccaagtcaaacttccccccaagcccgaacgacacacccacacacaacc
	pGAPDH			GGTTGAAATGAATCGGCCGACGCTCGGTAGTCGGAAAGAGCCGGGACCGGCCGGCGAGCATAAACCGGACGCAGTAGGATGTCCTGCACGGGTCTTTTTGTGGGGTGTGGAGAAAGGGGTGCTTGGAGATGGAAGCCGGTAGAACCGGGCTGCTTGGGGGGATTTGGGGCCGCTGGGCTCCAAAGAGGGGTAGGCATTTCGTTGGGGTTACGTAATTGCGGCATTTGGGTCCTGCGCGCATGTCCCATTGGTCAGAATTAGTCCGGATAGGAGACTTATCAGCCAATCACAGCGCCGGATCCACCTGTAGGTTGGGTTGGGTGGGAGCACCCCTCCACAGAGTAGAGTCAAACAGCAGCAGCAACATGATAGTTGGGGGTGTGCGTGTTAAAGGAAAAAAAAAGAAGCTTGGGTTATATTCCCGCTCTATTTAGAGGTTGCGGGATAGACGCCGACGGAGGGCAATGGCGCCATGGAACCTTGCGGATATCGATACGCCGCGGCGGACTGCGTCCGAACCAGCTCCAGCAGCGTTTTTTCCGGGCCATTGAGCCGACTGCGACCCCGCCAACGTGTCTTGGCCCACGCACTCATGTCATGTTGGTGTTGGGAGGCCACTTTTTAAGTAGCACAAGGCACCTAGCTCGCAGCAAGGTGTCCGAACCAAAGAAGCGGCTGCAGTGGTGCAAACGGGGCGGAAACGGCGGGAAAAAGCCACGGGGGCACGAATTGAGGCACGCCCTCGAATTTGAGACGAGTCACGGCCCCATTCGCCCGCGCAATGGCTCGCCAACGCCCGGTCTTTTGCACCACATCAGGTTACCCCAAGCCAAACCTTTGTGTTAAAAAGCTTAACATATTATACCGAACGTAGGTTTGGGCGGGCTTGCTCCGTCTGTCCAAGGCAACATTTATATAAGGGTCTGCATCGCCGGCTCAATTGAATCTTTTTTCTTCTTCTCTTCTCTATATTCATTCTTGAATTAAACACACATCAACA

	pOLE1			ttctattgtggaactcgtctctacctgtaggctctacttgtagctacaacaatagtgtgagaggagttgaaatccagctgagaacaccgtctctccgggattcacatccttagtctgctcacatcgagtgttagtctgct<br>cggaatcatctaacagccattttatagtccgttaatcctccatcataccgtataactgttagttagctacaagtatagtacgtatggtacacctccttctcccttctcacctccttctcccttctcaactccttctcaactcct<br>tctcaactccttctcaactccttctcgcctccttctcgcctccttctcctcctcacctccttctcacctcctctaacctctaacctccaacctcaaccctcatcctctactactcgctagactatgtcaatacactcaatcaca<br>acagtcgcattacaagaggatatggccgaaacctggacattgaactccgtaataagtccatcttccccatcctccatttgtcctcagctaatccattttttttcttctgactgcacagccgcgttcgcccttcgccttcgcc<br>tttgcctttgtgtttttttccccttgtccgactcgcagggaaaaaaatattaaactttttgtgtagaacatcttcttcatatgaaattgcccttctggcgtgccacatgcatagctctcaacgaatagacagcgcggatta<br>cgacgacccacgacggcggggcggtaatggcacgtgttcgtgtccgccaaaacgcccccacagcgcccaacccgcgccgccggcggttttccgccgtggcaaacaccgcaattgcgccaatggcaccgaaaccc<br>aactctcaacaagtcaaacctaaaaagcccaacaaatgcatgtgagatggaaggagcccaggcacactggtgctccaaccgcccttatatagatgggccgcttcccctacgtaatcgaatacttgtctttcctcatc<br>tactaactacacattcaac
	YlOLE1	YALI0C05951g	YALI1_C07638g	atggtgaaaaacgtggaccaagtggatctctcgcaggtcgacaccattgcctccggccgagatgtcaactacaaggtcaagtacacctccggcgttaagatgagccagggcgcctacgacgacaagggccgcca<br>catttccgagcagcccttcacctgggccaactggcaccagcacatcaactggctcaacttcattctggtgattgcgctgcctctgtcgtcctttgctgccgctcccttcgtctccttcaactggaagaccgccgcgtttgc<br>tgtcggctattacatgtgcaccggtctcggtatcaccgccggctaccaccgaatgtgggcccatcgagcctacaaggccgctctgcccgttcgaatcatccttgctctgtttggaggaggagctgtcgagggctccat<br>ccgatggtgggcctcgtctcaccgagtccaccaccgatggaccgactccaacaaggacccttacgacgcccgaaagggattctggttctcccactttggctggatgctgcttgtgcccaaccccaagaacaagggc<br>cgaactgacatttctgacctcaacaacgactgggttgtccgactccagcacaagtactacgtttacgttctcgtcttcatggccattgttctgcccaccctcgtctgtggctttggctggggcgactggaagggaggtct<br>tgtctacgccggtatcatgcgatacacctttgtgcagcaggtgactttctgtgtcaactcccttgcccactggattggagagcagcccttcgacgaccgacgaactccccgagaccacgctcttaccgccctggtcac<br>ctttggagagggctaccacaacttccaccacgagttcccctcggactaccgaaacgccctcatctggtaccagtacgaccccaccaagtggctcatctggaccctcaagcaggttggtctcgcctgggacctccaga<br>ccttctcccagaacgccatcgagcagggtctcgtgcagcagcgacagaagaagctggacaagtggcgaaacaacctcaactggggtatccccattgagcagctgcctgtcattgagtttgaggagttccaagagc<br>aggccaagacccgagatctggttctcatttctggcattgtccacgacgtgtctgcctttgtcgagcaccaccctggtggaaaggccctcattatgagcgccgtcggcaaggacggtaccgctgtcttcaacggaggt<br>gtctaccgacactccaacgctggccacaacctgcttgccaccatgcgagtttcggtcattcgaggcggcatggaggttgaggtgtggaagactgcccagaacgaaaagaaggaccagaacattgtctccgatgag<br>agtggaaaccgaatccaccgagctggtctccaggccacccgggtcgagaaccccggtatgtctggcatggctgcttag
	AaOLE1	OLE1 from *Arxula adeninivorans*		ATGAACGGTCCCGAAGAGGTGAATCTCGAAGAAGTCCAGGCCATTGCGTCTGGAGCCGAAGTTCGAGCTAAGGTCAACATCAACCGACGAAGGCAAGAGGAG<br>CAGGCTGCCGCCGCTGCTGCCTCCAGCGGTTCGACAAAGACTCATATCTCCGAGCAGGCTTTCACCCTCGCCAACTGGCACAAGCATTTCAATTGGATCAACACA<br>ACCATCATTGCCATTATCCCAGCAATCGGCTTTTTGTCGGTGCCTTTCATTCCTGTGCACGGCAAGACATTAGCGTGGGCATTCGTCTACTACTTTTTGACCGGTC<br>TGGGAATCACCGCCGGATACCACCGTCTGTGGGCTCACCGGGCTTACAGTGCATCATGGCCCCTGCGAGTGTTCTTGGCACTTTTGGGTGCTGGAGCCGGTGAG<br>GGTTCAGTAAAGTGGTGGTCTAATGGACACCGCACTCACCACCGTTACACTGACACTGACAAGGATCCTTACAATGCCAAGCGAGGATTCTGGTTCTCCCACAT<br>GGGCTGGATGATGTTCAAGCAGAACCCCAAGCTCAAGGGACGATGCGACATTTCCGATCTTATCTGCGACCCTATTATTCGATGGCAGCACCGACACTACATTT<br>GGATCATGGCAGCAATGTCGTTTGTATTCCCTTCTGTAGTTGCTGGACTGGGCTGGGGAGACTACCTGGGAGGATTTGTGTTTGCAGGAATCCTGCGACAGTTT<br>GTTGTCCACCAGTCGACCTTCTGTGTCAACTCGCTTGCCCACTGGCTGGGAGAGCAGCCTTTTGACGACAACCGATCTCCTCGAGACCACGTTCTGACTGCGTTT<br>GCTACTCTGGGTGAGGGTTACCACAACTTCCACCACGAGTTCCCCTCCGACTACCGTAACGCTATCAAGTGGTACCAGTACGACCCTACCAAGATCTTCATCTGG<br>ACCATGAAGCAGCTTGGTCTGGCCTCTAACCTGCAGACTTTCTCCCAGAATGCTATTGAGCAGGGTCTGGTACAGCAGAAGCAGAAGAAGCTGGACCGATGGA<br>GAGCTCGTCTCAACTGGGGAGTGCCTATTGAGCAGCTTCCTGTAATTGAGTACGACGACTTCAAGGACGAGTCATCTTCCCGATCTTTGGTCCTCATTTCTGGAA<br>TTGTCCACGATGTTACCGACTTTATTGACAAGCACCCTGGTGGAAAGGCTCTCATCAAGAGCGCCATTGGCAAGGACGGAACTGCCGTGTTCAACGGAGGTGTG<br>TACAAGCACTCCAACGCTGCTCACAACCTGCTGGCCACTATGCGTGTAGCTGTCATTCGAGGAGGAATGGAAGTCGAGGTCTGGAAGCGTGCCCAGGGCGAGA<br>AGAAGGATGTTGACCCTGTCGCCGATTCCGCTGGTGACCGTATCCTGCGAGCTGGCGACCAGCCTTCTCGTGTTCCTGAGGCCCGTGTCTCTGGCCGCGCTGCTT<br>AA

Gene

RtOLE1	OLE1 from *Rhodotorula toruloides*	ATGACTGCCTCTTCGGCACTCGAGACCTCGCTCCCGCACTCTGTCGGGCCCGAGGCTGCGACCACCACAGCAAAGCCGCCCCGTGCGCCGCTCAGGATGCGTCA<br>CCCCGACTACACTCAGACCGACGTCCTCGATTCGTCGGACTCGGATGCAGCGTCGGATTCTGAGGGCGAGACGACGGCGGTCGACGATGGGACCTACGAGGAC<br>GACAACTACGTCCGCAAGGTCCTCAGCAAGGAGAAGCCGCTCCCGCCCATCACCTGGAAGAACATCCACCGCAACATCCAGTGGATCTCGACCCTCGCCCTCAC<br>CATCGTGCCCCTCCTCGCCATCTACGGAGCGTTCACGACGCCCTTGAAGTGGCAGACGGCGGTCTGGAGTGTCGTCTACTACTACTACACCGGACTTGGTATCAC<br>GGCAGGCTACCACAGGCTGTGGGCCCACAGGTCCTACACCGCCTCTCTGCCTCTCCAGTACTTCTTGGCACTTGGAGGAAGCGGCGCAGTCGAGGGGAGCGTG<br>AAATGGTGGTCTAGGGGACACCGCGCACACCATCGCTACACCGACACAGACCTCGACCCGTATTCGGCGCAGAAGGGCTTCTGGTGGGCTCACCTTGGCTGGA<br>TGATCGTCAAGCCGCGCCGTCGTCCCGGTGTCGCCGACGTTTCCGACCTCAACAACAACCCGGTCGTCAAGTGGCAGCACCGCTACTATCTCCCGCTCATCCTCG<br>GCATGGGCTTCGTCTTCCCTACCATCGTCGCTGGACTCGGCTGGGGCGACTTCCGCGGCGGATTCTTCTTCGCCGGCGCCGCTCGCCTCCTCTTCGTCCACCACTC<br>GACGTTCTGCGTCAACTCGCTTGCGCACTGGCTGGGCGAGACGCCCTTCGACGACAAGCACACGCCGAAGGATCACTGGCTCACGGCGCTCGCGACTGTCGGT<br>GAGGGCTACCACAACTTCCACCACGAGTTCCCCTCCGACTACCGCAACGCACTTCGATGGTGGCAGTACGACCCGACTAAGCTCTTCATCTGGACGATGTCGAA<br>GCTCGGATTGGCGTCGCAGCTCAAGACGTTCCCCGACAACGAGATCAAGAAGGGCCAGTACGCGATGACGCTCAAGGCTGTCGCGCGCGAGGCCGAGAACAT<br>CGAGTGGCCCAAGTCGTCGAACCATTTGCCTGTGCTCACTTGGGACGAGTTCCAGGACGCCTGCAAGACCCGTCAGCTCCTTGTTGTCGCCGGTTTCATCCACGA<br>TGTCAGCACGTTCATCGACCAGCACCCCGGCGGTGCCGGCTTGATCAAGACTCGTCTCGGTCGCGACGCGACGACTGCCTTCTACGGTGGCTACTACGACCACT<br>CGAACGGCGCAGCCAACCTGCTCGCCCAGTACCGTGTCGGTGTCATCGAAGGCGGCTACGAGGTCGAGCACATGAAGAAGTATTCGGAGGTCGTCGAGAACCT<br>CAAGAAGCACGGCGCCGATGGCGTGGCCGGCAAGAGCGCCGACCTCGTCAAGGGCCCGAAGCAGACGTCGGTCATCAAGGGCGACCCTCAGCTGAAGAGCG<br>CGCCGCTCGAGACGCTCGCCAAGCCGCCCACCTTCAGCGAAACCAACCTCTTGGGCGGTCTCAGCCTGAAGGTCAAGGCGTAA
GtOLE	OLE1 from *Gloeophyllum trabeum*	ATGGCGACCTACACGCCGCCGCTGACGCCGCCGTCCGAGCCCACCAAGCGGCTCAAAAATCTCGAGCCGGAGCCCATCGACATCAACATCCCCGACAACTACGT<br>CCAGCACACGCTCAAGACGCAGAAGGAGCTCCCGCCTATCACCTGGAGCAACTGGTACCGCGAGCTGCAGTGGATCAGCGTCCTCGCGCTCACGATCACACCT<br>GCACTTGCGATCTACGGCGCGTTTACGACCAAGCTCACATGGCAGACGGGCCTCTTCAGCGTCTTCTACTACTACGTGACTGGCCTCGGTATCACCGCCGGATAC<br>CACCGTCTGTGGGCGCACCGGTCGTACAACGCCTCCAAGCCGTTGCAGTACTTCCTCGCGCTCGCGGGCTCGGGCGCCGTCGAGGGCTCCATCAAATGGTGGTC<br>GCGCGGCCACCGTGCGCACCATCGCTACACCGACACCGAACTCGACCCGTACTCGGCCCAGAAGGGCTTCTGGTGGTCGCACGTCGGCTGGATGCTCTTCAAGC<br>CGCGCCGCAAGCCCGGCGTCGCCGACGTCAGCGACCTCAGCCGCAACGAGGTCGTCAGGTGGCAGCACCGCTGGTATGTCTGGCTCATCCTTGGCATGGGCTT<br>CGGGCTCCCGACTGTTGTCCCGGGCCTGCTCTGGGGTGACTGGTGGGGCGGGTTCTTCTACGCGGGTGCTCTGCGCCTGACGTTCGTGCACCATTCGACGTTCT<br>GTGTCAACTCCCTAGCGCACTGGCTGGGAGAGACGCCGTTCGACGACAAGCACACGCCCAGGGACCACGTTATCACCGCGCTCGTCACCATCGGCGAGGGCTA<br>CCACAACTTCCACCACCAGTTCCCCATGGACTACCGCAACGCCATCAAGTGGTACCAGTACGACCCGACAAAGTGGTTCATCATGGCCTGCCAGTGGGTCGGCC<br>TCGCGTCACATCTCAAGACGTTCCCTGATAACGAAGTCCGCAAAGGCCAGCTGACTATGCAGCTCAAGCGGCTCCGGGAAACGCAGGAGAAGCTCACCTGGGC<br>ACCGGATAGCAACGACCTGCCCATTGTCTCTTGGGACAGCTTCCAGGAACAGTCGGCGAAGCGTCCGCTGATCTTGATTGCTGGCTTCATCCACGATGTCGCGT<br>CCTTCTTGGACGAGCACCCTGGCGGCCGGCATCTGCTCGTGAAGTACATCGGCAAGGACGCTACGACGGCGTTCTTCGGCGGTGTCTACGATCACTCCAACGCC<br>GCGCATAACCTGCTCTCGATGCACCGCGTTGGCATCCTGCAACACGGCTACCGCCAGAGCTTGGACGATAAGGCCATCCCGCCCGCGCAGCGCCTGCGGATTGC<br>GCGGTACAACGAGCTCGGGTCATCGACGGCCGTGTCGGATGCGGAGACGCTAGTGGGGGAGAAGGAGGAGAAGGAAGCGTAG

	FAD2	YALI0B10153g	YALI1_B13699g	atggattcgaccacgcagaccaacaccggcaccggcaaggtggccgtgcagcccccacggccttcattaagcccattgagaaggtgtccgagcccgtctacgacacctttggcaacgagttcactcctccagact<br>actctatcaaggatattctggatgccattccccaggagtgctacaagcggtcctacgttaagtcctactcgtacgtggcccgagactgcttctttatcgccgtttttgcctacatggcctacgcgtacctgcctcttattc<br>cctcggcttccggccgagctgtggcctgggccatgtactccattgtccagggtctgtttggcaccggtctgtgggttcttgcccacgagtgtggccactctgctttctccgactctaacaccgtcaacaacgtcaccgg<br>atgggttctgcactcctccatgctggtcccttactacgcctggaagctgacccactccatgcaccacaagtccactggtcacctcacccgtgatatggtgtttgtgcccaaggaccgaaaggagtttatggagaaccg<br>aggcgcccatgactggtctgagcttgctgaggacgctcccctcatgaccctctacggcctcatcacccagcaggtgtttggatggcctctgtatctgctgtctaacgttaccggacagaagtaccccaagctcaacaa<br>atgggctgtcaaccacttcaaccccaacgccccgctgtttgagaagaaggactggttcaacatctggatctctaacgtcggtattggtatcaccatgtccgtcatcgcatactccatcaaccgatggggcctggcttc<br>cgtcaccctctactacctgatcccctacctgtgggtcaaccactggctcgtggccatcacctacctgcagcacaccgaccccactctgccccactaccacgccgaccagtggaacttcacccgaggagccgccgcca<br>ccatcgaccgagagtttggcttcatcggctccttctgcttccatgacatcatcgagacccacgttctgcaccactacgtgtctcgaattcccttctacaacgcccgaatcgccactgagaagatcaagaaggtcatgg<br>gcaagcactaccgacacgacgacaccaacttcatcaagtctctttacactgtcgcccgaacctgccagtttgttgaaggtaaggaaggcattcagatgtttagaaacgtcaatggagtcggagttgctcctgacggc<br>ctgccttctaaaaagtag
	DGA1	YALI0E32769g	YALI1_E38810g	(ATG)ACTATCGACTCACAATACTACAAGTCGCGAGACAAAAACGACACGGCACCCAAAATCGCGGGAATCCGATATGCCCCGCTATCGACACCATTACTCAACC<br>GATGTGAGACCTTCTCTCTGGTCTGGCACATTTTCAGCATTCCCACTTTCCTCACAATTTTCATGCTATGCTGCGCAATTCCACTGCTCTGGCCATTTGTGATTGCG<br>TATGTAGTGTACGCTGTTAAAGACGACTCCCCGTCCAACGGAGGAGTGGTCAAGCGATACTCGCCTATTTCAAGAAACTTCTTCATCTGGAAGCTCTTTGGCCGC<br>TACTTCCCCATAACTCTGCACAAGACGGTGGATCTGGAGCCCACGCACACATACTACCCTCTGGACGTCCAGGAGTATCACCTGATTGCTGAGAGATACTGGCC<br>GCAGAACAAGTACCTCCGAGCAATCATCTCCACCATCGAGTACTTTCTGCCCGCCTTCATGAAACGGTCTCTTTCTATCAACGAGCAGGAGCAGCCTGCCGAGCG<br>AGATCCTCTCCTGTCTCCCGTTTCTCCCAGCTCTCCGGGTTCTCAACCTGACAAGTGGATTAACCACGACAGCAGATATAGCCGTGGAGAATCATCTGGCTCCAA<br>CGGCCACGCCTCGGGCTCCGAACTTAACGGCAACGGCAACAATGGCACCACTAACCGACGACCTTTGTCGTCCGCCTCTGCTGGCTCCACTGCATCTGATTCCAC<br>GCTTCTTAACGGGTCCCTCAACTCCTACGCCAACCAGATCATTGGCGAAAACGACCCACAGCTGTCGCCCACAAAACTCAAGCCCACTGGCAGAAAATACATCTT<br>CGGCTACCACCCCCACGGCATTATCGGCATGGGAGCCTTTGGTGGAATTGCCACCGAGGGAGCTGGATGGTCCAAGCTCTTTCCGGGCATCCCTGTTTCTCTTAT<br>GACTCTCACCAACAACTTCCGAGTGCCTCTCTACAGAGAGTACCTCATGAGTCTGGGAGTCGCTTCTGTCTCCAAGAAGTCCTGCAAGGCCCTCCTCAAGCGAAA<br>CCAGTCTATCTGCATTGTCGTTGGTGGAGCACAGGAAAGTCTTCTGGCCAGACCCGGTGTCATGGACCTGGTGCTACTCAAGCGAAAGGGTTTTGTTCGACTTG<br>GTATGGAGGTCGGAAATGTCGCCCTTGTTCCCATCATGGCCTTTGGTGAGAACGACCTCTATGACCAGGTTAGCAACGACAAGTCGTCCAAGCTGTACCGATTC<br>CAGCAGTTTGTCAAGAACTTCCTTGGATTCACCCTTCCTTTGATGCATGCCCGAGGCGTCTTCAACTACGATGTCGGTCTTGTCCCCTACAGGCGACCCGTCAACA<br>TTGTGGTTGGTTCCCCCATTGACTTGCCTTATCTCCCACACCCCACCGACGAAGAAGTGTCCGAATACCACGACCGATACATCGCCGAGCTGCAGCGAATCTACA<br>ACGAGCACAAGGATGAATATTTCATCGATTGGACCGAGGAGGGCAAAGGAGCCCCAGAGTTCCGAATGATTGAGTAA
